# Mitochondria and Brain Disease: A Comprehensive Review of Pathological Mechanisms and Therapeutic Opportunities

**DOI:** 10.3390/biomedicines11092488

**Published:** 2023-09-07

**Authors:** Vicente Javier Clemente-Suárez, Laura Redondo-Flórez, Ana Isabel Beltrán-Velasco, Domingo Jesús Ramos-Campo, Pedro Belinchón-deMiguel, Ismael Martinez-Guardado, Athanasios A. Dalamitros, Rodrigo Yáñez-Sepúlveda, Alexandra Martín-Rodríguez, José Francisco Tornero-Aguilera

**Affiliations:** 1 Faculty of Sports Sciences, Universidad Europea de Madrid, Tajo Street, s/n, 28670 Madrid, Spain; vctxente@yahoo.es (V.J.C.-S.); josefrancisco.tornero@universidadeuropea.es (J.F.T.-A.); 2 Group de Investigación en Cultura, Educación y Sociedad, Universidad de la Costa, Barranquilla 080002, Colombia; 3 Department of Health Sciences, Faculty of Biomedical and Health Sciences, Universidad Europea de Madrid, C/Tajo s/n, Villaviciosa de Odón, 28670 Madrid, Spain; 4 Psychology Department, Facultad de Ciencias de la Vida y la Naturaleza, Universidad Antonio de Nebrija, 28240 Madrid, Spain; 5 LFE Research Group, Department of Health and Human Performance, Faculty of Physical Activity and Sport Science-INEF, Universidad Politécnica de Madrid, 28040 Madrid, Spain; 6 Department of Nursing and Nutrition, Faculty of Biomedical and Health Sciences, Universidad Europea de Madrid, 28670 Villaviciosa de Odón, Spain; pedro.belinchon@universidadeuropea.es; 7 Facultad de Ciencias de la Vida y la Naturaleza, Universidad de Nebrija, 28015 Madrid, Spain; imartinezgu@nebrija.es; 8 Laboratory of Evaluation of Human Biological Performance, School of Physical Education and Sport Sciences, Aristotle University of Thessaloniki, 54124 Thessaloniki, Greece; dalammi9@hotmail.com; 9 Faculty of Education and Social Sciences, Universidad Andres Bello, Viña del Mar 2520000, Chile; rodrigo.yanez.s@unab.cl

**Keywords:** mitochondria, brain disease, neurodegenerative disorders, mitochondrial DNA mutations, oxidative phosphorylation, mitochondrial dynamics, reactive oxygen species, mitochondrial protective agents

## Abstract

Mitochondria play a vital role in maintaining cellular energy homeostasis, regulating apoptosis, and controlling redox signaling. Dysfunction of mitochondria has been implicated in the pathogenesis of various brain diseases, including neurodegenerative disorders, stroke, and psychiatric illnesses. This review paper provides a comprehensive overview of the intricate relationship between mitochondria and brain disease, focusing on the underlying pathological mechanisms and exploring potential therapeutic opportunities. The review covers key topics such as mitochondrial DNA mutations, impaired oxidative phosphorylation, mitochondrial dynamics, calcium dysregulation, and reactive oxygen species generation in the context of brain disease. Additionally, it discusses emerging strategies targeting mitochondrial dysfunction, including mitochondrial protective agents, metabolic modulators, and gene therapy approaches. By critically analysing the existing literature and recent advancements, this review aims to enhance our understanding of the multifaceted role of mitochondria in brain disease and shed light on novel therapeutic interventions.

## 1. Introduction

Mitochondria, the energy-producing powerhouses within cells, have emerged as crucial players in the pathogenesis of brain diseases. The intricate relationship between mitochondrial dysfunction and neurodegenerative disorders, psychiatric illnesses, and other brain-related conditions has garnered significant attention in scientific research. Understanding the role of mitochondria in brain disease is essential for unravelling the underlying mechanisms, identifying potential therapeutic targets, and improving patient outcomes [1].

In this line, neurodegenerative disorders, including Alzheimer’s disease, Parkinson’s disease, Huntington’s disease, and amyotrophic lateral sclerosis, are characterised by the progressive loss of neuronal function and structure. A growing body of evidence suggests that mitochondrial dysfunction contributes significantly to the onset and progression of these debilitating conditions. Mitochondrial DNA mutations, which disrupt normal cellular function, have been implicated in the development of neurodegenerative disorders, further highlighting the critical role of mitochondria in brain health. Impaired oxidative phosphorylation, the primary energy-generating process within mitochondria, has been implicated in the pathophysiology of brain diseases. Dysregulation of this essential metabolic pathway leads to compromised ATP production, resulting in energy deficits and neuronal dysfunction. Such impairments have been observed in various brain disorders, underscoring the significance of maintaining mitochondrial function for neuronal health [2].

In relation to mitochondrial dynamics, the processes governing mitochondrial fission, fusion, and transport have recently gained attention in the context of brain disease. Aberrant mitochondrial dynamics have been linked to neuronal damage and cell death, highlighting the importance of maintaining a balanced mitochondrial network for optimal neuronal function. Moreover, calcium dysregulation, a hallmark of brain disease, can directly impact mitochondrial function, leading to detrimental consequences for neuronal viability. In addition, while moderate levels of reactive oxygen species (ROS) serve as signaling molecules involved in cellular processes, excessive production can result in oxidative stress and mitochondrial dysfunction. Imbalance in the delicate redox homeostasis within neurons contributes to neuronal damage and neurodegeneration [3].

In line with oxidative stress, inflammation, another common feature of brain diseases, has been closely associated with mitochondrial impairment. Inflammatory mediators, such as cytokines and chemokines, can induce mitochondrial dysfunction through various mechanisms. For instance, the production of nitric oxide (NO) and ROS during inflammation can directly damage mitochondrial components, including lipids, proteins, and DNA. This oxidative damage compromises mitochondrial function, leading to reduced ATP production and impaired cellular energy metabolism [4].

Furthermore, inflammation-induced alterations in calcium homeostasis can disrupt mitochondrial dynamics and promote mitochondrial fragmentation. Calcium influx into mitochondria can trigger excessive mitochondrial fission, impairing mitochondrial function and exacerbating energy deficits. Additionally, inflammatory signalling pathways can activate pro-apoptotic proteins, leading to mitochondrial outer membrane permeabilization and the subsequent release of pro-inflammatory molecules, such as cytochrome c, which further propagates the inflammatory response. Conversely, mitochondrial dysfunction can activate immune responses and contribute to the perpetuation of disease pathology. Dysfunctional mitochondria release damage-associated molecular patterns (DAMPs) and mitochondrial DNA (mtDNA) fragments, which can act as danger signals and trigger innate immune responses. Activation of pattern recognition receptors (PRRs) by these mitochondrial-derived molecules leads to the production of pro-inflammatory cytokines and chemokines, perpetuating the inflammatory state in the brain. This reciprocal relationship between inflammation and mitochondrial dysfunction forms a vicious cycle in brain diseases. Chronic inflammation perpetuates mitochondrial impairment, while mitochondrial dysfunction promotes sustained inflammation. This cycle further amplifies neuronal damage, exacerbates neurodegenerative processes, and impairs the brain’s ability to mount effective immune responses [5].

In relation to psychiatric illnesses, neurotransmitters, such as serotonin, dopamine, and glutamate, play crucial roles in regulating mood, cognition, and overall brain function. Emerging evidence suggests that dysregulation of mitochondrial function can influence neurotransmitter metabolism and signalling, contributing to the pathogenesis of psychiatric disorders. Mitochondrial dysfunction can disrupt the balance of neurotransmitters through multiple mechanisms. For example, impaired oxidative phosphorylation and reduced ATP production can affect the synthesis, release, and reuptake of neurotransmitters. Additionally, mitochondrial dysfunction can lead to oxidative stress, which can directly damage neurotransmitter receptors and transporters, impairing their function. Altered mitochondrial dynamics and impaired calcium homeostasis can also impact neurotransmitter release and synaptic transmission [6].

Depression, bipolar disorder, and schizophrenia are psychiatric disorders that have been associated with mitochondrial dysfunction and dysregulated neurotransmitter systems. In depression, mitochondrial impairment in the prefrontal cortex and hippocampus, brain regions involved in mood regulation, has been observed. Similarly, abnormalities in mitochondrial function and energy metabolism have been identified in individuals with bipolar disorder. Dysregulation of neurotransmitters, such as dopamine and glutamate, has been implicated in the pathophysiology of both depression and bipolar disorder. In schizophrenia, alterations in mitochondrial function, including impaired energy metabolism and oxidative stress, have been reported. The dysregulation of neurotransmitters, particularly dopamine and glutamate, is also a prominent feature in schizophrenia and is associated with the development of psychotic symptoms [7].

Given the critical role of mitochondria in brain disease, numerous therapeutic strategies have been explored to protect, identify biomarkers, and restore mitochondrial function. Mitochondrial protective agents, such as antioxidants and mitochondrial-targeted compounds, have shown promise in preclinical and clinical studies. Additionally, metabolic modulators that can restore energy metabolism and enhance mitochondrial function are being investigated as potential therapeutic interventions. Recent advances in gene therapy have opened up exciting possibilities for restoring mitochondrial function in brain diseases. By targeting specific mitochondrial genes or introducing exogenous genetic material, gene therapy holds the potential to repair mitochondrial defects and mitigate disease progression. Thus, understanding the mechanisms underlying mitochondrial dysfunction and exploring novel therapeutic strategies targeting mitochondrial health are crucial steps towards advancing our knowledge and improving patient outcomes in various brain disorders. The subsequent sections of this manuscript will delve into the specific aspects of mitochondrial dysfunction in brain diseases, highlighting the current state of research and potential avenues for therapeutic interventions [8].

### Methodology of Search

A systematic approach was employed to gather the relevant literature for this narrative review on neurodegenerative disorders and mitochondrial dysfunction. A comprehensive search was conducted using primary and secondary sources, including scientific articles, bibliographic indexes, and databases such as PubMed, Scopus, Embase, Science Direct, Sports Discuss, ResearchGate, and the Web of Science. To ensure the relevance of the gathered literature, MeSH-compliant keywords were utilised. Keywords such as Mitochondria AND brain disease; Neurodegenerative disorders AND mitochondrial dysfunction; Mitochondrial DNA mutations AND brain disease; Impaired oxidative phosphorylation AND brain disease; Mitochondrial dynamics AND neuronal health; Calcium dysregulation AND mitochondria; Reactive oxygen species AND mitochondrial dysfunction; Inflammation AND mitochondrial impairment AND brain disease; Mitochondria AND neurotransmitter systems AND psychiatric illnesses; Mitochondrial biomarkers AND brain disease; Therapeutic strategies AND mitochondrial protective agents; Metabolic modulators AND mitochondrial function AND brain diseases; Gene therapy AND restoring mitochondrial function; were employed to retrieve articles that align with the specific objectives of this study.

The search period was limited to articles published between July 2003 and July 2023, ensuring the currency and pertinence of the included data. The titles and abstracts of all retrieved manuscripts were meticulously examined by the review authors to determine their suitability for analysis. Exclusion criteria were applied to filter out studies that utilised outdated data, focused on unrelated topics, or were not written in the English language. Upon identifying relevant studies, the review authors independently extracted information from the selected articles. This rigorous approach helps maintain the quality and reliability of the data included in the review. Furthermore, collaborative discussions were conducted among the review authors to synthesise the findings and present a comprehensive narrative that addresses the specific objectives of the study. By pooling their expertise and insights, the review authors ensure a cohesive and informative analysis of the literature.

## 2. Molecular Mechanisms in the Development of Diseases

Mitochondria are essential for normal cellular function because they are responsible for energy production in eukaryotes, as well as phospholipid and heme synthesis, calcium homeostasis, apoptotic activation, and cell demise [1,9]. As a comprehensive overview of multiple pathological processes, the following will analyse and identify the molecular processes occurring in the mitochondria that are involved in specific common patterns in the pathological process’s development. Mitochondrial diseases are a group of genetic disorders characterised by defects in oxidative phosphorylation and caused by mutations in genes that encode structural mitochondrial proteins or proteins involved in mitochondrial function in nuclear DNA (nDNA) and mitochondrial DNA (mtDNA). Mitochondrial diseases are the most prevalent category of inherited metabolic disorders and one of the most prevalent types of inherited neurological diseases [10,11]. During normal neuronal development, programmed cell death (PCD) signalling events occur in a spatially and temporally restricted manner to establish the neural architecture and shape the CNS. In the pathogenesis of numerous neurological diseases, abnormalities in PCD signalling cascades, such as apoptosis, necroptosis, pyroptosis, ferroptosis, cell death associated with autophagy, and unprogrammed necrosis, can be observed. These cell fatalities can be triggered in response to intracellular or extracellular stimuli and inflammatory processes. A prevalent characteristic of neurodegenerative diseases such as amyotrophic lateral sclerosis (ALS), Alzheimer’s disease, Parkinson’s disease, and Huntington’s disease is the aberrant activation of PCD pathways, which leads to the unwarranted loss of neuronal cells and function [12].

### 2.1. Mitochondria Bioenergetics

The mitochondria contain the main enzymatic systems required to complete the oxidation of sugars, lipids, and proteins to produce adenosine triphosphate (ATP), which can be used for energy production. Each of these three substrates can be catabolized to acetyl-CoA, which then enters the citric acid cycle in the mitochondrial matrix, the first of these processes [1]. Following glycolysis in the cytosol, glucose enters the mitochondria as pyruvate. Pyruvate dehydrogenase aids in the conversion of pyruvate to acetyl-CoA. Inside the mitochondria, beta oxidation converts fatty acids to acetyl-CoA, while various enzymes convert specific amino acids into pyruvate, acetyl-CoA, or directly into specific citric acid cycle intermediates [13].

In the citric acid cycle, also known as the tricarboxylic acid (TCA) or Krebs cycle, the acetyl group of acetyl-CoA is transferred to oxaloacetate to form the six carbon molecule citrate. In seven successive enzymatic steps, citrate is oxidised back to oxaloacetate, with the excess carbon removed as two molecules of carbon dioxide and the electrons transferred to cofactors nicotinamide adenine dinucleotide (NADH) and flavin adenine dinucleotide (FADH2). The oxaloacetate is now free to rejoin the cycle, while NADH and FADH2 transport the liberated energy to the mitochondrial electron transport chain [14].

The electron transport chain, also known as the respiratory chain, is comprised of a series of multisubunit protein complexes embedded in the interior mitochondrial membrane [15]. The electrons removed from the citric acid cycle by NADH and FADH2 are used to enable the pumping of protons from the matrix to the intermembrane space, thereby generating a potential difference across the inner mitochondrial membrane [16]. In the final phase of oxidative phosphorylation, this potential difference is ultimately used to fuel the synthesis of ATP [15].

By binding to the largest of the respiratory complexes, NADH dehydrogenase, or complex I, NADH provides free energy to the electron transport chain. NADH transfers a pair of electrons, which are transported away from the citric acid cycle, to a flavin mononucleotide prosthetic group located inside the hydrophilic arm of complex I. Subsequently, the electrons are sent up the arm through a sequence of iron-sulphur clusters until they reach the lipid-soluble redox carrier known as coenzyme Q [9].

The transfer of four protons across the inner mitochondrial membrane is associated with the transport of electrons from NADH via the complex. NADH must diffuse into complex I in order to furnish the electrons it delivers to electron transport. Conversely, succinate dehydrogenase, the enzyme responsible for catalysing the reduction of FAD to FADH2 in the citric acid cycle, is an integral component of the electron transport chain. Commonly referred to as complex II, this enzyme with a molecular weight of 123 kDa is situated on the inner mitochondrial membrane, similar to complex I [17]. It consists of FAD as a prosthetic group in addition to iron-sulphur clusters, which facilitate the transfer of donor electrons to coenzyme Q. This complex does not transport any protons from the mitochondrial matrix. It is noteworthy that this particular complex is distinct from other respiratory chain complexes in that it is only encoded by nuclear DNA [9].

The reduced form of coenzyme Q may traverse the inner mitochondrial membrane without hindrance and afterwards transfer its electrons to cytochrome c reductase, the third complex of the electron transport chain. This reduction can occur via either complex I or complex II. The final outcome of the electrons transferred through the electron transport chain is the conversion of oxygen into water. This phenomenon takes place in the fourth complex, known as cytochrome c oxidase [18].

During the progression of electrons along the electron transport chain, there is a gradual reduction in their free energy, which coincides with a consistent rise in the redox potential of the carriers involved. Ultimately, this process culminates with oxygen, possessing the highest redox potential among all the carriers. The energy that is liberated as the electron moves down the free energy “staircase” serves as the driving force for the thermodynamically unfavourable process of proton pumping against their concentration gradient at complexes I, III, and IV [9].

After the completion of the citric acid cycle and the electron transport chain, the final step in converting the energy stored in substrate molecules into ATP, which serves as the universal “energy currency”, involves the linkage of the membrane voltage of approximately 200 mV to the phosphorylation of adenosine diphosphate (ADP). In addition to causing the decoupling of membrane voltage from ATP generation, proton leakage is also accompanied by the leakage of electrons from the complexes of the electron transport chain [15]. The premature release of electrons enables their direct transfer to oxygen, bypassing the conventional pathway of electron transfer to oxygen for water formation at complex IV and therefore generating superoxide. Superoxide has a high level of reactivity, rendering it significantly detrimental to cellular function by inducing what is commonly referred to as “oxidative stress” [19]. The involvement of oxidative stress has been suggested in several pathological conditions, ranging from atherosclerosis and diabetes to neurodegenerative disorders and cancer. Additionally, it is widely believed that oxidative stress significantly contributes to the process of ageing [20,21]. For instance, the association between AD and oxidative stress has been extensively established as a result of bioenergetic processes. ROS are employed as cell-signalling molecules at low concentrations. ROS can have detrimental effects in conditions characterised by diminished levels of antioxidants and impaired redox processes, as shown in ageing and instances of mitochondrial malfunction [22].

### 2.2. Calcium Signalling

Mitochondria are in constant communication with the cytosol in order to coordinate the equilibrium between the energy needs of the cell and the energy produced by oxidative phosphorylation [9]. This is orchestrated predominantly by calcium signalling between the cytosol and matrix. Ca^2+^ signalling is essential for the majority of cellular ‘activation states’: Ca^2+^ signals regulate the majority of processes that are associated with increased energy demands—secretion, contraction, motility, and electrical excitability—all of which necessitate an increase in energy provision and are typically accompanied by an increase in cytosolic Ca^2+^ concentration ([Ca^2+^]c) [23,24]. In the inner mitochondrial membrane, mitochondria exhibit a Ca^2+^ uptake pathway, the mitochondrial calcium uniporter (MCU), which is a Ca^2+^ selective channel. As Ca^2+^ moves down its electrochemical potential gradient into the matrix, a rise in local [Ca^2+^]c stimulates mitochondrial Ca^2+^ uptake [25]. The increase in matrix Ca^2+^ concentration ([Ca^2+^]m) stimulates the three rate-limiting enzymes of the TCA cycle: pyruvate, α-ketogluterate (also known as oxogluterate), and NAD-isocitrate dehydrogenases. A rise in [Ca^2+^]m also appears to upregulate ATP synthase, although the mechanism remains unknown. Together, these mechanisms increase the supply of NADH to the respiratory chain, respiration, and ultimately the rate of ATP synthesis. The increase in [Ca^2+^]c also activates the glutamate/aspartate transporter (ARALAR) on the inner mitochondrial membrane, thereby increasing substrate supply; this pathway does not require specific mitochondrial Ca^2+^ uptake and is therefore independent of mitochondrial bioenergetic competence [24]. Thus, these pathways function together to elegantly and simply match energy supply and demand. In the majority of cells, the efflux of Ca^2+^ from the mitochondrial matrix, driven by a Na^+^/Ca^2+^ exchanger, is relatively sluggish, such that the change in [Ca^2+^]m significantly outlasts the change in [Ca^2+^]c, and the metabolic response likely matches the time course of the change in [Ca^2+^]m. At least in muscle, where exercise is associated with an increase in mitochondrial biogenesis mediated by Ca^2+^, the coupling of energy supply and demand occurs on a prolonged timescale [23]. Emerging research suggests that there is an aberration in neuronal calcium signalling in several neurodegenerative conditions, including Alzheimer’s disease (AD), Huntington’s disease (HD), and Parkinson’s disease (PD) [23].

### 2.3. Cell Death

Significant evidence supports a role for cell death in the pathogenesis of numerous brain and peripheral nervous system diseases. However, it is still unclear whether defects in cell death signalling and neuronal cell death are a primary or secondary response to the attacks that cause these diseases, as well as how different PCD pathways and additional processes interact to cause the death of neuronal cells and other cell types in these diseases [12,26].

The regulation of the intrinsic route involves the modulation of pro- and anti-apoptotic members belonging to the BCL-2 protein family [27]. The preservation of cell viability in healthy cells is facilitated by a group of proteins known as anti-apoptotic proteins, namely BCL-2, BCL-XL, MCL-1, BCL-W, and A1/BFL1 [28]. These proteins fulfil this role by inhibiting the crucial mediators of cell death, BAX and BAK. The BH3-only proteins, including BIM, PUMA, BID, BMF, BAD, HRK, BIK, and NOXA, which play a crucial role in initiating apoptosis, are seen to undergo transcriptional or post-transcriptional upregulation in reaction to intracellular stress such as growth factor deprivation, DNA damage, and ER stress [28]. The BH3-only proteins have a strong binding affinity for anti-apoptotic BCL-2 proteins, resulting in the release of BAX and BAK. Several studies have demonstrated that some BH3-only proteins have the ability to directly activate BAX and BAK. Upon being activated, BAX and BAK proteins undergo oligomerization, leading to mitochondrial outer membrane permeabilization (MOMP). This process results in the release of cytochrome c and Smac/DIABLO from the mitochondria. The apoptogenic factors facilitate the initiation of the caspase cascade, which subsequently triggers the cleavage of many proteins, ultimately culminating in cellular implosion [12]. The available evidence supporting the involvement of apoptosis in neuronal cell death in AD is constrained. Nevertheless, according to the immuno-histochemical labelling of neurons, it has been postulated that intracellular Aβ has the potential to trigger apoptosis via the p53-dependent transcriptional upregulation of BAX [29]. Additionally, the maximum expression of BAX in the brain was seen in grade 2 and 3 Huntington’s disease (HD) brains, as described in previous studies [30].

Necroptosis is a kind of programmed cell death characterised by cellular lysis, which has the potential to induce an inflammatory response. Necroptosis may be triggered by the activation of TNFR1, TLRs, and specific additional receptors in instances when the function of caspase-8 is impeded by pharmaceutical substances or viral inhibitors. The mechanism under consideration entails the activation of receptor-interacting serine/threonine protein kinase 1 (RIPK1) by autophosphorylation [12]. The reduction of amyloid load, inflammatory cytokine levels, and memory problems was shown in a mouse model of Alzheimer’s disease with the implementation of pharmacological inhibition or genetic deletion of RIPK1 [31].

Additionally, one characteristic feature commonly observed in neurodegenerative illnesses is the presence of proteinaceous aggregates and ubiquitinated inclusion bodies. These aggregates and inclusion bodies are believed to have a role in the development and progression of these diseases [32]. The presence of abnormal autophagy is a characteristic observed in several neurological disorders. Amyotrophic lateral sclerosis (ALS) is characterised by the presence of genetic mutations in autophagy-related genes, including SQSTM1, OPTN, TBK1, VCP, and C9ORF72, which have been found to be linked to family variants of the illness. It has been discovered that there is an increase in autophagosomes in the cytoplasm of spinal cord neurons in patients with amyotrophic lateral sclerosis (ALS) [33].

## 3. Neurodegenerative Disorders and Mitochondrial Dysfunction

**Figure 1:**
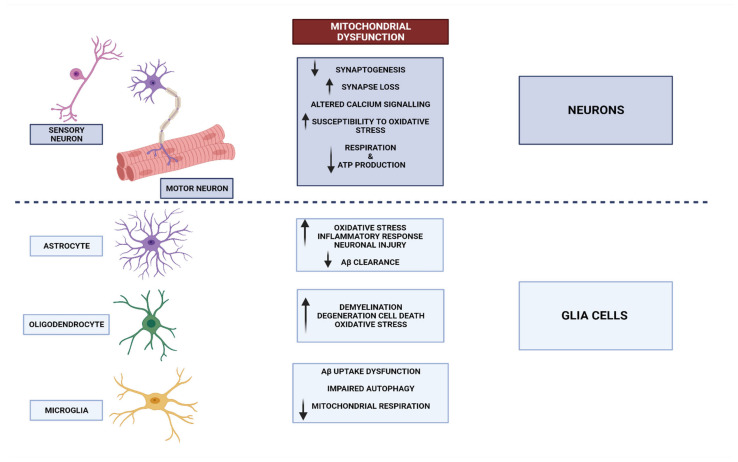
Consequences of mitochondrial dysfunction depending on glia cells or neuron types [37,62,63].

The information transmitted by neurons is crucial to normal brain operation [34]. Neurons can be found in many other tissues and organs beyond the brain [35]. Glia, also known as neuroglia, constitute a significant proportion of cellular entities within the intricate framework of the nervous system [36]. While neurons exhibit excitability and play a crucial role in information processing, glial cells take on the complete responsibility for maintaining homeostasis and defending the central nervous system (CNS). Various types of glial cells have been implicated in both normal and pathological brain function [37]. Astrocytes elegantly extend their processes throughout grey matter, gracefully enveloping synapses with their presence. These remarkable cells play a pivotal role in maintaining the delicate balance of ions and neurotransmitters, ensuring a harmonious environment within the central nervous system. Furthermore, they diligently oversee the intricate process of synaptogenesis, orchestrating the formation of vital connections between neurons. It is worth noting that astrocytes also possess the remarkable ability to secrete various substances, thus earning them the esteemed title of secretory cells within the CNS [38]. Microglia are responsible for regulating the elimination of apoptotic neurons and play a role in influencing the formation of synaptic connections during brain development [39]. In pathological conditions, these microglia become activated and contribute to the occurrence of neuroinflammation and myelination, which play a crucial role in shaping the connectome. The essential glial functions, as well as the interaction between glial cells and neurons, delineate the functioning of the brain in both states of well-being and pathology [37]. In recent decades, significant advancements have been made in comprehending the pivotal role of glial cells in the advancement of neural pathologies, encompassing neurodevelopmental disorders, neurodegeneration, and demyelinating pathologies (Figure 1) [40,41].

Neural stem cells generate most neurons during development, but their numbers drastically fall once we reach adulthood [42]. Although neuronal death is inevitable, neurodegeneration remains a major health issue because of its role in the pathophysiology of certain neurological disorders [43]. Neurodegeneration is associated with the accumulation of proteins with physiochemical modifications, synapse dysfunction, and neural network malfunction [44]. Related to this, neurodegenerative disorders (NDs) are the medical terminologies used to describe diseases that exhibit the characteristics of neurodegeneration. Some of the most common NDs seen in clinical praxis are Parkinson’s disease, Alzheimer’s disease, prion disease, amyotrophic lateral sclerosis, motor neuron disease, spinal muscular atrophy, Huntington’s disease, and spinocerebellar ataxia [45,46].

In this context, it is enlightening to contemplate Alzheimer’s disease, Parkinson’s disease, amyotrophic lateral sclerosis, and Huntington’s disease as illustrative instances. Considering the diverse range of these ailments, it is highly probable that mitochondrial involvement serves as a pivotal connection among them [47]. In addition to its vital role in generating ATP, this remarkably dynamic organelle also regulates processes such as apoptosis, ferroptosis, and inflammatory activation, all of which play crucial roles in ensuring cellular survival [48]. The preservation of cell viability, both in states of wellness and illness, hinges upon the criticality of the mitochondrial response to various stresses. This is primarily due to the multifaceted nature of the roles undertaken by mitochondria. Recent scientific investigations have unveiled the crucial involvement of mitochondria in the initiation and advancement of neurodegenerative conditions [49].

### 3.1. Mitochondrial Dysfunctional Functioning in Neurodegenerative Disorders

The phenomenon known as mitochondrial biogenesis refers to the intricate process wherein fresh mitochondria are engendered through the growth and division of pre-existing mitochondria. This intricate process ultimately leads to a notable augmentation in the mitochondrial mass within a given cell [50]. Protein synthesis from the mitochondrial and nuclear genomes, as well as replication of mitochondrial DNA (mtDNA), are all part of this process, which is regulated by peroxisome proliferator-activated receptor gamma (PPAR γ) and PGC1 α [51]. PGC1 α functions as a master regulator that integrates and coordinates the activity of nuclear respiratory factors 1 and 2 (NRF1-2) and mitochondrial transcription factor A (TFAM) [52]. By attaching to TFAM promotor sites, they set off the mitochondrial transcription and replication processes [52,53]. The reduction in ATP synthesis, compromise in mitochondrial integrity, and elevation in oxidative stress are all consequences observed when these processes become imbalanced [54].

**Figure 2:**
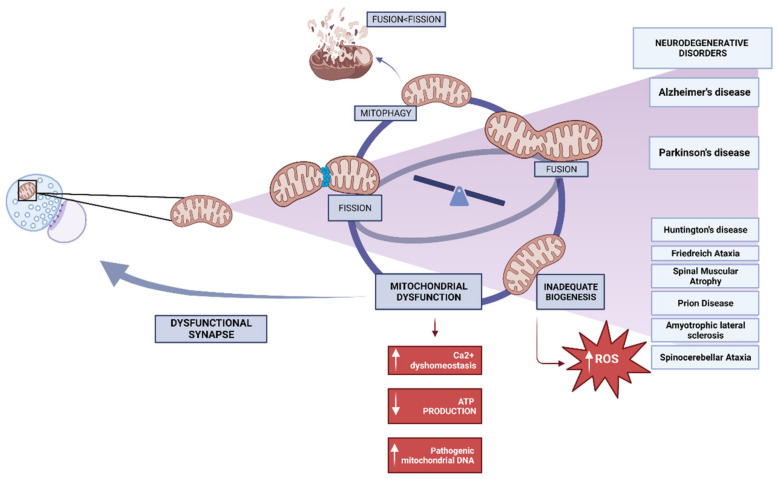
Mitochondrial dysfunction in biogenesis processes and dynamics triggers neurodegenerative diseases.

Deficiencies in the process of mitochondrial biogenesis may potentially contribute to the manifestation of mitochondrial dysfunction in various neurodegenerative conditions, such as Alzheimer’s disease, Parkinson’s disease, Huntington’s disease, and Friedreich ataxia, among other notable examples [55]. The neurodegenerative process is believed to involve oxidative stress from ROS generation, but the downstream steps of mitochondrial malfunction leading to neuronal cell death are not well known. Mitochondria are both the main generators of ROS and the principal targets of increased exposure to them. Due to elevated synthesis and decreased clearance, ROS exposure increases when mitochondrial biogenesis is inadequate (Figure 2) [56].

Moreover, with the progression and increased accessibility of imaging technologies, the previously held notion of mitochondria as stationary, bean-shaped cellular structures has been invalidated. The mitochondria, being highly mobile and dynamic organelles, exhibit a remarkable ability to undergo fusion and fragmentation. This intricate process, referred to as “mitochondrial dynamics”, is tightly regulated and occurs in response to various environmental stimuli [57]. In a cell, mitochondria that have fused together can be seen as a tubular network with many connections. The efficient transfer of mtDNA during mitochondrial biogenesis and a metabolic rate that matches the cell’s energy needs are both made possible by this network. Consequently, the health of a cell depends on a finely tuned balance between fusion and fission (Figure 2). A homeostatic mechanism that allows the cell to effectively adjust to rapidly fluctuating energy demands, mitochondrial dynamics also ensures the quality of mitochondria [58,59]. The disruption of mitochondrial dynamic processes can result in abnormal mitochondrial morphology and function [58]. Mitochondrial morphology dysfunction results in decreased respiratory and bioenergetic capabilities in cells. When there is a high concentration of pathogenic mtDNA, these bioenergetic functions are utterly lost. Mitochondrial division and fusion influence the distribution of mitochondrial DNA [60]. Furthermore, the mitochondria in fragmented cells have a lower membrane potential and generate less ATP via the electron transport chain. Changes in the function or structure of mitochondria can have severe consequences for neurons since they are essential to neuronal function. Mitochondria break apart and contribute to brain injury when fission is favoured over fusion [61].

The focus of scientific inquiry pertaining to mitochondrial dysfunction and its implications in neurological disorders has predominantly revolved around neurons. On the contrary, the involvement of malfunctioning mitochondria in the functioning of glial cells and its potential impact on neuronal homeostasis and brain function have received limited attention in the scientific literature. However, based on the available evidence, it is evident that the maintenance of mitochondrial health within glial cells plays a crucial role in the proper functioning of neurons [62] (Figure 1).

#### 3.1.1. Mitochondrial Dysfunction in Alzheimer’s Disease

Abnormalities in mitochondrial function, such as elevated ROS generation, decreased ATP synthesis, disturbed mitochondrial Ca^2+^ homeostasis, and mitochondrial dynamics and transport abnormalities, are strongly linked to Alzheimer’s disease. For instance, mitochondrial malfunction and calcium homeostasis disturbances might lead to synaptic dysfunction. Neuronal mitochondrial dysfunction is exacerbated by these physiologic changes. Some details of AD, the most common form of neurodegenerative disease, remain unknown. Amyloid plaques, made of amyloid-beta protein (A), and neurofibrillary tangles, made of hyperphosphorylated tau, are seen extracellularly in AD patients [63,64]. Aβ induces apoptotic cell death in neurons via mechanisms involving oxidative stress, inflammation, and mitochondrial dysfunction, like those characteristics associated with mitochondrial biogenesis deficiencies. This data supports the hypothesis that defective mitochondrial biogenesis contributes to the aetiology of AD. Decreased levels of peroxisome proliferator-activated receptor coactivator 1 (PGC1 α), nuclear factor erythroid 2-related factor 2 (Nrf2), and mitochondrial transcription factor A (TFAM) have been seen in cellular and murine models of AD and in postmortem brain tissue of AD patients [65,66]. In both cellular and mouse models of AD, pharmacological activation, or overexpression of PGC1 α protects against nitrosative stress, increases mitochondrial mass as evidenced by increased citrate synthase (CS) activity and OXPHOS levels, reverses mitochondrial dysfunction, and decreases neuronal loss [67]. Furthermore, brain-derived neurotrophic factor (BDNF) is one of the most extensively distributed neurotrophins implicated in synaptogenesis and neuroprotection, and PGC1 α regulates its expression [68]. The BDNF levels in the brains of AD patients decreased. In the APP/PS1 mouse model of Alzheimer’s disease, Aβ decreases brain BDNF expression, but BDNF treatment inhibits Aβ deposition and ameliorates cognitive decline [69]. In this regard, Pohland et al. (2018) pointed out that pharmacological activation of PGC1 α in Thy-1 APPSL rodents restores mitochondrial membrane potential, ATP levels, and mitochondrial mass and increases BDNF levels [67]. These findings imply that defective PGC1 α mediated signalling may contribute on multiple levels to the pathology of Alzheimer’s disease.

Thus, mitochondrial dysfunction and oxidative damage have been extensively studied for their potential role in the aetiology of AD. Before severe plaque pathology develops, oxidative damage occurs in the AD brain [70]. Reddy’s laboratory has conducted extensive research on mitochondrial dysfunction in Alzheimer’s disease and has published numerous articles describing both the manifestations and causes of mitochondrial dysfunction in Alzheimer’s disease. For instance, they showed that the upregulation of genes involved in mitochondrial metabolism and apoptosis occurs significantly earlier and co-localises with the neurons experiencing oxidative damage in transgenic APP mice [30], suggesting that oxidative damage precedes A β deposition in these mice [71]. Additionally, Swerdlow’s reported evidence suggests that mtDNA may be implicated in the mitochondrial dysfunction observed in Alzheimer’s disease. When patient mtDNA is transferred into mtDNA-deficient cell lines, the resulting ‘hybrids’ reproduce the respiratory enzyme deficiency observed in the brain and other tissues of Alzheimer’s disease, suggesting that mtDNA abnormalities are at least partially responsible for the defect [72].

Although mitochondrial microRNA science is emerging, it is still relatively new in the mitochondrial and synapse sections of Alzheimer’s disease research. It is well known that miRNAs serve crucial roles in mitochondrial function [73]. Those seeking to develop biomarkers, treatments, and/or targets for the treatment of Alzheimer’s disease are naturally more interested in mitochondrial microRNAs [74,75]. However, little is known about mitochondrial microRNAs that affect specific synaptic functions. No miRNA has been linked to neurotransmission-relevant processes such as mitochondrial trafficking, Ca^2+^ signalling, or synaptic vesicle formation [75]. In addition, only a handful of mitochondrial miRNAs were linked to synaptic activity, neurotransmission, synaptic plasticity, and neurotoxicity [76]. Identifying additional mitochondrial miRNAs in the synapse associated with these functions and determining whether or not they are dysregulated in Alzheimer’s disease will provide a greater understanding of which miRNA should be the primary focus when analysing the synaptic dysfunction present in Alzheimer’s disease [77].

#### 3.1.2. Mitochondrial Dysfunction in Parkinson’s Disease

Parkinson’s disease (PD), the second most prevalent neurodegenerative disorder, is characterised by symptoms including bradykinesia, resting tremor, postural instability, akinesia, and occasionally dementia. The disease is characterised by a range of mitochondrial abnormalities, including impairment of the electron transport chain, changes in mitochondrial morphology and dynamics, mutations in mitochondrial DNA, and disturbances in calcium homeostasis. These abnormalities are depicted in Figure 1 [78]. From a pathological standpoint, these characteristics are indicative of a more pronounced decline in dopaminergic (DA) neurons and the existence of Lewy bodies containing α -synuclein in the substantia nigra pars compacta (SNpc). Additionally, there is an observed impairment in the levels of striatal dopamine in individuals with PD [79]. There are several genes associated with Parkinson’s disease that suggest a potential involvement of mitochondria in the development and progression of the disease. Thus far, it has been elucidated that mutations or polymorphisms in mtDNA as well as a minimum of nine specifically designated nuclear genes have been recognised as culprits behind the onset of Parkinson’s disease (PD) or influencing the susceptibility to PD. The patient presents with a list of proteins and molecules that are of medical significance. These include α-synuclein, parkin, ubiquitin carboxy-terminal hydrolase L1, DJ-1, phosphatase, and tensin homologue (PTEN)-induced kinase 1 (PINK1), leucine-rich-repeat kinase 2 (LRRK2), the nuclear receptor NURR1, HTRA2, and tau. The nuclear genes α-synuclein, parkin, DJ-1, PINK1, LRRK2, and HTRA2 are intricately associated with mitochondrial processes, either directly or indirectly [47,80]. Additionally, reduced activity of respiratory chain complex I (NADH: ubiquinone oxidoreductase) suggests deficits in mitochondrial biogenesis contribute to the pathogenesis of Parkinson’s disease in multiple models, including 1-methyl 4-phenyl-1,2,3,6-tetrahydropyridine (MPTP)’s effects on dopaminergic (DA) neurons [81]. Decreased complex I activity is also seen in patients with parkin and PINK1 mutations, the two most common causes of autosomal recessive familial PD [82]. Mitochondrial biogenesis has also been linked to mitochondrial malfunction in Parkinson’s disease through other lines of evidence. Patients with PD have lower levels of PGC1 α, and mutations in this gene are linked to an earlier age of onset and an increased risk of PD42 [83]. PGC1 α responsive genes are also underexpressed in dopaminergic neurons microdissected from PD patients.

In summary, the findings indicate that the presence of complex I deficiency, heightened oxidative stress, and mutations in mitochondrial DNA collectively contribute to the pathogenesis of Parkinson’s disease [78]. The potential causes of mitochondrial injury in sporadic PD remain elusive. Mitochondrial dysfunction and the subsequent development of Parkinson’s disease can potentially arise because of environmental factors, including exposure to pesticides. This correlation has been observed in both human subjects and animal models used to study the disease [84]. Furthermore, it is important to note that familial mutations in genes can also give rise to mitochondrial dysfunction, which in turn can lead to the development of Parkinson’s disease. In patients diagnosed with PD and in animal models, it has been observed that there are certain general mitochondrial biochemical abnormalities. These abnormalities primarily manifest as impaired functioning of complex I, a crucial component of the mitochondrial respiratory chain [85], decreased ATP synthesis [86], increased ROS production, mitochondrial DNA mutations [87], and defective mitochondrial repair pathways. However, the chain of events leading to mitochondrial impairment and neuronal degeneration remains obscure [78]. Furthermore, the studies suggest that dopaminergic neurons located in the substantia nigra exhibit a higher vulnerability to stress compared to other neuronal classifications. The patient presents with a condition characterised by an elevated oxidative burden during dopamine metabolism, excitotoxicity, and an increased iron content [88], and low mitochondrial mass in substantia nigra dopaminergic neurons relative to other neurons may contribute to their selective vulnerability under stress conditions [89].

#### 3.1.3. Mitochondrial Dysfunction in Other Neurodegenerative Disease

Huntington’s disease (HD) is an autosomal dominant neurodegenerative disorder characterised by involuntary movements, psychiatric disturbances, and subcortical dementia [90]. It is caused by an expansion of CAG repeats that confers a toxic function on the Huntingtin (Htt) protein [91]. Although HD’s neuropathology manifests itself in multiple brain regions, it is most obviously seen in the striatum, where it is defined by selective cell death. Although the processes are unknown, defects in mitochondrial biogenesis may contribute to the striatum’s susceptibility to Huntington’s disease. Transcriptional sup-pression by mHtt-dependent sequestration of transcription factors CREB/TAF4 has been found in the striatum of HD mouse models and human patients, leading to lower levels of PGC1 α mRNA and protein [92,93]. Oxidative stress, such as lipid peroxidation and DNA oxidative damage, is significantly elevated in HD, thereby amplifying the formation of amyloidogenic mutant huntingtin (mHtt) aggregates and possibly contributing to the vicious cycle in the pathophysiology of HD [94]. Regarding Friedreich ataxia (FRDA), it is the most prevalent form of autosomal recessive ataxia and is characterised by degeneration of large sensory neurons and spinocerebellar tracts, cardiomyopathy, and an increased incidence of diabetes. It is caused by an expanded GAA triplet repeat in the first intron of the FXN gene, which results in transcriptional suppression and a decrease in the expression of frataxin, a mitochondrial protein essential for iron-sulphur cluster biogenesis [95]. In model systems, a reduction in frataxin expression decreases the activity of iron-sulphur cluster-containing enzymes such as mitochondrial respiratory chain complexes and aconitase, resulting in mitochondrial iron accumulation, ATP deficiency, and oxidative stress [96].

## 4. Mitochondrial DNA Mutations in Brain Disease

The mitochondria have the characteristic that their cellular function is regulated by a double energy system. On the one hand, it has nuclear DNA that it shares with the rest of the cells of the organism, and on the other hand, it has DNA of its own, the mtDNA, which is composed of thirty-seven genes, thirteen peptides, two ribosomal RNAs, and twenty-two ribonucleic acids, or transfer RNAs [97]. It is precisely this unique origin of mtDNA from the egg that explains why mitochondrial pathologies present maternal patterns in the exclusive alterations of mtDNA [98]. It is known that the main function of mitochondria is to facilitate cellular respiration for metabolic energy, in addition to other functions involved in pathways such as cytogenesis, thermogenesis, intracellular Ca2 regulation, or fatty acid oxidation [99]. Therefore, mitochondrial diseases mean a very high rate of mutations that will be transmitted generation after generation since there are very few mechanisms for repair and protection of mitochondrial DNA [100].

Although evidence has been found that there are mutations in genes encoded in nDNA, it has been established in recent decades that the main mutations in mtDNA lead to dysregulation and malfunction of the oxidative phosphorylation system [101]. All these mutations cause the so-called mitochondrial pathologies or mitochondrial encephalomyopathies, still considered “rare diseases” since their prevalence is low, i.e., less than 5/10,000 inhabitants [102,103]. However, the impact on the paediatric population is very serious, this being the most frequent age range of onset. Similarly, diagnosis in adults is becoming more common [104]. These diseases are still a challenge for medicine today and are characterised by very heterogeneous clinical manifestations with alterations in different tissues and organs. However, they share the alteration of oxidative phosphorylation with the presence of encephalopathy and lesions at the muscular level [105,106]. All mitochondrial diseases have in common a deficiency in the biosynthesis of adenosine triphosphate (ATP) that causes the cells to have less energy and symptoms associated with this alteration, which will depend on the cellular regions affected [107,108]. In this sense, the first disease that appeared associated with mutations in mtDNA was Leber’s hereditary optic neuropathy, although later studies were able to relate this pathology to 16 mutations [109].

In relation to the brain pathologies that have been determined so far to be associated with mitochondrial mutations, we find symptomatology such as seizures, convulsions, and reduced intellectual capacity, although the symptomatology will depend on the phenotype, the mutation, and the level of complementation with normal mtDNA [110,111,112]. Mitochondriopathies present a wide variety of phenotypes, depending on their association with point mutations or when insertions, deletions, or both appear and the DNA is restructured. Among those with point mutations, neuropathy, ataxia, and retinopathy pigmentosa syndrome, or NARP, a pathology whose clinic is characterised by the presence of neurogenic muscle weakness due to the m.8993 T > C/G mutation with heteroblastic transition, is presented in [113,114,115].

Leber’s hereditary optic neuropathy is included in this categorisation and is associated with bilateral optic atrophy and other symptoms such as dystonia. Early onset (12–30 years) and more prevalent in males than in females [116]. The most frequently identified mutations are A-G 11,778 in the ND4 gene and G-A 3460 in the ND1 gene [117,118]. Another disease associated with point mutations is Myoclonic Epilepsy and Ragged Red Fibers Syndrome (MERRF), produced by an A-G 8344 mutation and a reduced number of T-C 8356 [119]. Mitochondrial encephalomyopathies, lactic acidosis, and encephalic stroke-like episodes syndrome, or MELAS, are related to the A-G 3243 mutation [120].

In relation to diseases due to insertions or deletions, or both, that produce restructuring of mtDNA, the Kearns–Sayre syndromes, Pearson syndromes, as well as the Leukoencephalopathic syndromes and chronic sporadic progressive external ophthalmoplegia are related [121]. In this line, a recent study has been able to identify psychiatric and neurodegenerative pathologies and diseases associated with ageing related to genetic alterations in the mitochondrial DNA of the brain [122]. This study refers to difficulties in the methodology of previous studies, which focus on a single type of mutation or use obsolete techniques and provide little objective and quantitative data, and is a starting point for understanding the associated brain diseases [123]. This study includes, in addition to those mentioned above, neurological diseases with different neurodegenerative disorders such as AD and PD as the most frequent, although other phenotypes such as amyotrophic lateral sclerosis (ALS), Creutzfeldt–Jakob disease, Lewy body dementia, hippocampal sclerosis, Down’s syndrome, or mild cognitive impairment (MCI), among others, are also presented [121,124]. In all of them, the most recurrent alterations are mtDNA deletions and mtDNA variants [125,126].

In AD and PD, the results indicated elevated levels of heteroplasmy as well as a higher presence of variants in the parietal cortex, cerebellum, and hippocampus in AD patients compared to the control group. Specifically in AD, the most frequent mutation is the 4977 bp deletion (m.8470_13477del) in the temporal lobe, which occurs with a higher mutation load in the caudate nucleus than in the frontal gyrus [127]. In addition, this mutation load is found in the frontal cortex, substantia nigra, and globus pallidus at reduced levels (less than 2%) [127,128]. In PD, it has been possible to demonstrate that the existence of mtDNA mutation loads in MT-CO1, MT-CO2, and MT-CYB in the substantia nigra pars compacta, as well as in MT-CYB in the frontal cortex, was higher than in patients in the control group. Likewise, a significant increase in the level of heteroplasmy is described in neurons with a high number of deletions [127,128,129].

In relation to psychiatric disorders, we have mainly studied Schizophrenia (SZ) and bipolar disorder (BD), although we have also identified studies on psychiatric pathologies— Autism Spectrum Disorders (ASD)—caused by the consumption of alcohol or other substances [122,130]. Studies in SZ identify a frequency <0.02%), such as m.6617 C > T p. Phe238 (MT-CO1), m.8881T > C p.Ser119Pro (MTATP6), and m.9500C > T p.Phe98, m.9699A > G p.Ile165Val, and m.9956A > G p.Leu250 (MT-CO3). Isolated studies show deletion in the m.3243A>G variant and a 60% mutation load in m.12027T > C, p.Ile423Thr (MT-ND4) [131,132,133]. In BD, the synonymous variant m.10858T > C in the MT-ND4 gene has been identified in one case study and the variant m.3243A > G in two other patients, with low frequency [134,135].

Understanding the pathological mechanisms of mitochondrial DNA mutations should be useful to design effective therapeutic interventions in each patient, knowing the heterogeneity of the clinical presentation [136]. Currently, there is no treatment for these pathologies, with some exceptions in which the mutation appears in a single organ and is amenable to transplantation [137]. Different techniques and protocols for gene therapy are currently available, but none allow the correction of mutations in mtDNA. The rest of the measures used in these patients have a palliative function [138,139].

In this line, several studies are underway on effective treatments for mitochondrial pathologies. Among the most important are the selective inhibition of the propagation of mutant mtDNA, which is aimed at the introduction of enzymes that can recognise and cleave the mutant mtDNA parts in the mitochondrion; mitochondrial expression vectors, which are based on the introduction of complete or fragmented mtDNA with an original copy of the gene that can control the expression of mitochondrial genes, although this is not yet possible in practice; and allotopic expression, currently considered a viable option for the treatment of mitochondrial pathologies caused by mtDNA mutations. It is based on the introduction of genetic material into the cell nucleus through adenoviral vectors so that the protein produced could spread to all the mitochondria of the altered cell [140,141].

The mitochondrion is currently considered a therapeutic target that, in recent years, has caused great interest among the scientific and health communities. It has already been addressed in different pathologies, such as Charcot–Marie–Tooth disease, demonstrating that the avoidance of overproduction of ROS in the mitochondria with florfenicol as well as the prevention of oxidative damage with the antioxidant MitoQ prevent the progression of motor symptoms associated with this pathology as well as other cognitive and sensory symptoms [142]. This procedure is also being used in the treatment of alcoholic liver disease, which is responsible for up to 80% of liver-associated deaths and includes different pathological entities such as liver fibrosis, cirrhosis, simple steatosis, or hepatocellular carcinoma. In these diseases, the first lesion to manifest itself is the functional and structural modifications of the mitochondria, so liver-specific silencing of the MCJ protein was performed using RNA interference, which reduced the lesion and favoured the regeneration of the organ [143]. In this same vein, recent studies showed that certain activators of the mitofusin 2 protein could be therapeutic targets in type 2 diabetes. This protein is present in all cells and regulates multiple mitochondrial functions, so the decrease of this protein produces insulin resistance [144,145].

## 5. Impaired Oxidative Phosphorylation and Brain Disease

Cellular bioenergetics has studied cellular processes over the last few decades and has been able to confirm that most of these processes are induced by the hydrolysis of adenosine triphosphate (ATP). Through the prolonged oxidation of fatty acids, amino acids, and monosaccharides, ATP homeostasis is maintained [146]. The energy of these elements is transferred mainly as electrons to flavin adenine dinucleotide (FAD) or nicotinamide adenine dinucleotide (NAD+), although some are converted directly to ATP [99]. For the maintenance of the molecular oxidation process, the reoxidation of the reduced electron transporters is essential, which is mediated by the action of the enzyme lactate dehydrogenase (LDH) in the cytosol or by the action of the OXPHOS system in the mitochondrion. In addition, this process facilitates a higher energy yield [147].

The OXPHOS system ensures higher energy utilisation thanks to its dual activity. On the one hand, the electron transport chain includes ATP synthase, coenzyme Q (CoQ10), cytochrome c (Cytc), and the respiratory complexes CI-IV, proteins that are in the inner membrane of the mitochondrion and that are originated by the unification of units encoded in both the mtDNA and the DNA of the mitochondrial nucleus [101]. On the other hand, the propagation of protons from the mitochondrial matrix to the intermembrane space occurs and facilitates the emergence of the proton-motive force, essential for ATP synthesis. The last process in this system involves the utilisation of electrochemical energy to propagate ATP synthesis [148,149].

In this line, only certain proteins can effectively regulate the energy balance by identifying variations in the ratio of the levels of adenosine triphosphate, or ATP, adenosine diphosphate, or ADP, and adenosine monophosphate, or AMP [150]. These proteins are fructose-1,6-bisphosphatase, phosphofructokinase, and glycogen phosphorylase. In addition, the coenzyme NAD+ (nicotinamine adenine dinucleotide) is involved in mitochondrial metabolism, influencing different metabolic processes, but its most important role is related to deacetylation by sirtuins (seven members (SIRT1–7) that possess deacetylase activity), whose interest has been increasing in recent years [151,152,153]. Lines of research have been opened that relate the action of sirtuins with longevity and ageing processes [154].

Sirtuins are one of the primary metabolic sensors in the cell, and this is possible because they are more sensitive to metabolic state, increasing the NAD+/NADH (reduced nicotinamine adenine dinucleotide) ratio after physical activity or fasting, stimulating deacetylation by sirtuins [155,156]. In addition, the OXPHOS system is involved in other cellular actions such as the regulation of cytosolic calcium levels, the regulation of apoptosis, and the production of ROS, among others [157,158].

Regarding the energy metabolism of the cells of the Central Nervous System (CNS), neurons need a large amount of ATP and are dependent on the constant circulation of glucose [39,159]. In addition, these cells have no glycogen reserves. Therefore, it is important to maintain the functionality of the mitochondria in these cells since a deterioration of this functionality is related to neurodegeneration, both in old age and in diseases such as AD [160]. When there is an impairment in oxidative phosphorylation mediated by the OXPHOS system, the antioxidant mechanisms of the biological system are imbalanced, and this will lead to alterations in metabolic pathways and their activity, as well as the accumulation of intracellular aggregates, apoptosis, excitotoxicity, and mitochondrial dysfunction [161]. Oxidative stress is directly associated with molecular damage and neurodegenerative pathologies [162].

In this line, recent studies based on animal models, specifically an in vitro model of the neuroblastoma cell line SH-SY5Y, have been able to find neuronal loss in the substantia nigra leading to dopaminergic hypofunction [163]. It is also interesting to know that many AD patients present parkinsonian symptomatology as well as significant damage to the cholinergic system. The presence of significant cortical cholinergic denervation suggests that these cells are more vulnerable to pathology [163,164]. In Parkinson’s disease, or PD, one of the most prevalent pathologies worldwide in adults, degeneration of dopaminergic neurons of the substantia nigra pars compacta appears. In addition, these patients showed Lewy bodies in the cerebral cortex in postmortem studies [165]. In these patients, damage is found in the nigrostriatal pathway involved in the control of fine motility and is caused by a decrease in dopamine production. When there is increased oxidative stress, the different metabolic mechanisms carried out by dopamine can trigger membrane lipid peroxidation and cell death [166]. In addition, amino chromes can be formed, with a very high reactive power leading to O_2_ and the weakening of NADPH (Nicothioamide-Adenine Dinucleotide Phosphate) [167]. Finally, amino chromes are related to an increase in NO, O_2_, and H_2_O_2_ [168,169].

In Huntington’s disease (HD), alterations in oxidative phosphorylation occur due to increased aggregation of the mHTT protein, also known as the Huntingtin gene (HTT), whose main function is the regulation of the passage from DNA to mRNA for the formation of proteins [170,171]. The modification of the levels of this protein produces mitochondrial alterations [172]. In these patients, brain damage was found in cerebrospinal fluid (CSF), brain tissue, and lymphoblasts [172]. The damage produced by the deterioration of oxidative phosphorylation produces an increase in the concentration of malondialdehyde and 4-hydroxynonenal in the cerebral cortex and striatum [173]. In addition, a decrease in reduced glutathione was observed, as well as an increase in catalase and glutathione peroxidase [174].

There is also evidence that impaired oxidative phosphorylation is implicated in the aetiology of schizophrenia (SQ). In these patients, a higher level of lipid peroxidation products has been found, as well as a decrease in superoxide dismutase (SOD) activity, which evidences a clear mitochondrial dysfunction, causing metabolic alterations and neuronal deterioration [175,176]. Neuroplasticity is also impaired, which may affect cognitive functioning [177]. Studies in this line showed that certain genetic polymorphisms coding for enzymes with antioxidant function could be involved in a greater predisposition to the disease. They found significant differences in the genotypic profile of patients in the control group and patients with schizophrenia, with reduced values in the MnSOD gene (Ala-9Val polymorphism) [178,179]. On the other hand, the Pro-197Leu polymorphism of the glutathione peroxidase gene was analysed, although no significant findings were found between the group of patients with SQ and the control group [180,181].

In bipolar disorder (BD), lithium has been shown to reduce apoptosis processes in a cellular model, with 3β-GSK being a key mediator [182,183]. Significant differences in tau protein phosphorylation levels were also found in patients with this pathology. This is consistent with the physiological mechanisms of oxidative phosphorylation presented by people with neurodegenerative diseases, considered as “type 3 diabetes” or “brain diabetes” [184,185]. In addition, variations in mtDNA are associated with this disorder, indicating alterations in the capacity to regulate mitochondrial Ca++ [186]. The Ca++-mediated regulatory systems are altered, resulting in decreased Ca++ reuptake and increased intracytoplasmic Ca++ levels [187]. All this causes an increased sensitivity of the monoaminergic systems that originate the symptomatology associated with the disease [188]. The deterioration of mitochondrial function in specialised brain cells is very relevant since its involvement and bioenergetics in complex processes such as neuronal differentiation are of great importance.

## 6. Mitochondrial Dynamics in Neuronal Health

Neurons are complex, excitable, and polarised cells that require enormous amounts of energy. This demand for adenosine triphosphate (ATP) is primarily met through oxidative phosphorylation in mitochondria. Mitochondria play an essential role in neuronal function, undergoing constant cycles of fission/fusion at sites of energy demand. The morphology of mitochondria varies between axonal and dendritic compartments. Axons, which are smaller due to their self-correcting capacity that prevents extremes in size, exhibit more dynamic behaviour than dendrites. Recent advances have improved our understanding of mitochondrial dynamics and neuronal function. In particular, the mitochondrial permeability transition (MPT) governs mitochondrial dynamics and its transformation. Balanced fission/fusion is crucial for transportation. Moreover, defective mitochondrial function leads to the formation of impaired synapses, affecting neurite integrity and causing degeneration. Thus, impaired mitochondrial function emerges as a common thread in neurodegenerative disorders, and disordered fission/fusion tools serve as precursors in the development of pathophysiological mitochondrial function [189].

The knowledge of the importance of mitochondria for neuronal health is increasingly consolidated. In fact, mitochondria are essential for maintaining local energy supplies and attenuating calcium flux in neurons. Devine et al. established in their 2016 review the dependence of miro proteins on coordinating mitochondrial trafficking, dynamics, and turnover in neuronal homeostasis [190]. Specifically, mitochondrial dynamics are crucial for adjusting mitochondrial activity and efficiency. Initially, mitochondria were considered isolated and immobile organelles, but they are now recognised as highly active and interconnected systems within cells. This movement not only affects mitochondrial structure but also impacts the activity of organelles present in the cytoskeleton. This process, specifically regulated by mitochondrial fission/fusion cycles, is known as mitochondrial dynamics. These dynamics play a prominent role in cellular homeostasis, and abnormalities in mitochondrial fission/fusion proteins are associated with diseases, particularly age-related diseases. Altered expression of certain dynamic mitochondrial proteins is linked to ageing and age-related disorders, connecting ageing with a decline in mitochondrial activity and defective mitochondrial biogenesis. In this regard, age impairs key proteins in the autophagy mechanism, which, along with mitophagy, is responsible for eliminating defective mitochondria. Therefore, the combination of eliminating damaged mitochondria and proper mitochondrial biogenesis is key to maintaining a healthy mitochondrial balance [191].

In addition to ageing, various neurodegenerative disorders are determined by mitochondrial dysfunction. Avdoshina et al. [192] demonstrated that the gp120 protein of HIV reduces mitochondrial capacity and distribution. These alterations in mitochondrial distribution initiate neurodegeneration because the disruption of mitochondrial trafficking negatively affects energy distribution in synapses. It is noteworthy that these consequences occur even in the absence of the virus, suggesting that this protein alone is sufficient to initiate definitive neurodegenerative mechanisms, interacting with different endogenous neurotoxins or different pathophysiological insults [192]. In this line, the study of the role of proteins in neuronal mitochondrial dynamics continues to advance. HSPA9/mortalin is a mitochondrial chaperone protein considered a neuronal stress sensor and linked to the analysis of protein efficiency imported into the mitochondrial matrix. Therefore, the levels of neuronal mortalin expression are closely linked to mitochondrial stress control through modulation of axonal fragility. Ferré et al. [193] mechanically reported that mortalin overexpression is axo-protective. This quality correlates with its ability to increase mitochondrial length by acting on the activation of the mitochondrial fission protein DRP1. This again explains the scope of mitochondrial dynamics in the development of neurodegenerative diseases [193]. The level of mortalin is, therefore, crucial for cellular health. Seth, in his 2022 review, details that an excess of mortalin aids in the irregular proliferation observed in cancer cells, while inhibiting mortalin promotes oxidative stress, enhances mitochondrial functions, and reduces lifespan in pathologies such as AD, PD, and HIV. However, overexpression of mortalin has been proven to be a key factor in mitigating the consequences of AD, PD, and HIV in brain cells. Thus, a better understanding of the protective role of mortalin in neurodegenerative diseases is necessary [194].

As we have observed, studying the characteristics of mitochondrial dynamics, including fission/fusion, transport, biogenesis, and degradation, is essential for understanding neuronal health. In fact, mitochondrial dynamics and bioenergetics are interconnected. This is particularly relevant for neurons, as they depend on mitochondria for bioenergetic support. Van Laar and Berman [195] highlight the importance of the interplay between bioenergetics and mitochondrial dynamics in neurodegenerative processes. Different forms and structural characteristics of mitochondria correlate with the diverse bioenergetic needs of the tissues they occupy. Neurons rely on mitochondrial oxidative phosphorylation as their primary energy supply. Preserving a functional population of healthy mitochondria is therefore particularly challenging at the neuronal level. Indeed, PD has been linked to alterations in the regulation of mitochondrial dynamics. A deeper understanding of bioenergetics and mitochondrial dynamics in the pathophysiology of PD may lead to neuroprotective therapies in the early stages of the disease [195].

The ageing of the population has led to an increase in the prevalence of neurodegenerative diseases. In this regard, the scientific literature shows promising advances towards understanding the crucial role of mitochondrial dynamics in neuronal health. Therefore, research in this area can be crucial for reducing the disorders caused by neurodegenerative diseases.

## 7. Calcium Dysregulation and Its Impact on Mitochondria

In recent years, significant advances have been made in understanding alterations in Ca^2+^ homeostasis and mitochondrial degradation, highlighting the fundamental role of mitochondria in preventing apoptosis, especially in motoneurons (Jaiswal, 2014). A relevant function of synaptic mitochondria is to store and attenuate intracellular Ca^2+^, which regulates neurotransmission. The maximum capacity of Ca^2+^ absorption by mitochondria increases with more efficient fusion but decreases in fragmented mitochondria [196].

Cellular dysfunctions affecting structures such as mitochondria and the endoplasmic reticulum, along with increased oxidative stress and dysregulation of calcium homeostasis, are linked to the pathogenesis of Alzheimer’s disease (AD). The sodium-calcium exchanger (NCLX) is crucial for cellular metabolism regulation in both the plasma membrane and mitochondria. The dependency between energy metabolism and intracellular calcium levels is proposed as one of the first modifiable deteriorations in brain ageing. Therefore, it is essential to determine whether modifying NCLX activity to increase cellular metabolism and mitochondrial calcium content could prevent neuronal impairment and death [50]. Studies by Ludtmann and Abramov in 2018 on neurons deficient in the mitochondrial protein PINK-1 and neurons overexpressing alpha-synuclein suggest that NCLX may also be related to the pathophysiology of Parkinson’s disease (PD). Thus, mitochondrial Ca^2+^ and NCLX appear to be critical for therapeutic advancements in PD [197].

Both PD and AD are neurodegenerative diseases originating from misfolding and aggregation of key proteins. Oligomeric beta-amyloid and alpha-synuclein stimulate mitochondrial depolarization due to excessive calcium and free radical generation, leading to the opening of the mitochondrial permeability transition pore and ultimately cell death [198].

Among neurodegenerative diseases related to ageing, AD is the most prevalent and is associated with the deposition of beta-amyloid protein in senile plaques. Intracellular calcium acts as a second messenger and is essential for regulating neuronal functions, action potential, and synaptic plasticity. Abnormal aggregation of beta-amyloid peptide (Aβ) is proposed as a precursor to degenerative events in cholinergic neurons. Therefore, alterations in calcium homeostasis are investigated to understand the processes of Aβ-induced neurodegeneration. Neurofibrillary tangles (NFTs), aggregates of hyperphosphorylated tau protein, give rise to the “tau hypothesis”, where NFTs are considered the main culprits in neuronal loss and memory impairment due to axonal transport alterations in AD. The review by Cascela and Cecchi [199] reveals that there is no consensus regarding the molecular processes related to excessive neuronal Ca^2+^ overload, leading to signal transduction reconfiguration with excitotoxicity and memory reduction in AD. However, recent advances suggest that both Aβ and tau pathologies have synergistic consequences. Thus, the most appropriate therapy to slow down AD progression may involve combining anti-Aβ and anti-tau therapies [199]. Although much remains to be understood about the pathophysiology of AD regarding Ca^2+^ uptake, Jadiya et al. [200] suggested that enhancing the elimination of pathogenic Ca^2+^ could be a significant therapeutic benefit in reducing the progression of AD and other neurodegenerative diseases [200].

Scientific evidence indicates that excitatory synaptic dysregulation participates in neurodegeneration. In fact, dysregulation of mitochondrial calcium is associated with post-synaptic excitatory neurodegeneration. It was documented how excitatory lesions associated with neurodegeneration in PD, AD, amyotrophic lateral sclerosis (ALS), and HD. Therefore, therapeutic research should also focus on preserving mitochondrial function through calcium homeostasis regulation [201].

In this context, the primary excitatory neurotransmitter in the brain is glutamate, which binds to several receptors, including the N-methyl-D-aspartate receptor (NMDAR). NMDARs play roles in synaptic plasticity and excitotoxic cell death. Mitochondria, in turn, have fundamental functions such as ATP production and calcium regulation. Apoptosis, or the opening of the mitochondrial transition pore, resulting from disturbed mitochondrial calcium homeostasis, can lead to neuronal death. This undesirable outcome is related to the pathogenesis of diseases such as AD, PD, stroke, or traumatic brain injury. Therefore, the role of glutamate is indispensable for synaptic plasticity and cell death in relation to NMDA receptors, which are permeable to calcium and generate different signalling pathways depending on their location [201]. Calcium signalling is crucial for mitochondrial activity, and its dysregulation is linked to the development of neurodegenerative diseases such as AD. Dysregulated calcium homeostasis disrupts mitochondrial function, which is associated with neurodegeneration. According to Ryan et al. [202] review, understanding the mechanisms of mitochondrial calcium entry and function is critical, and the mitochondrial calcium uniporter complex (MCU) is proposed as a potential therapeutic target in neurodegenerative diseases [202].

In this context, the deficiency of coenzyme Q8a in purkinje neurons is the primary cause of cerebellar ataxia. Again, altered mitochondrial function, dysregulation of intracellular calcium, and altered dendrites are present in this ataxia. Therefore, the structure of purkinje neurons, altered mitochondrial function, and calcium dysregulation may be restored through treatment with coenzyme Q10 [203].

As observed, multiple elements participate in the pathophysiology of neurodegenerative diseases. The literature highlights the fundamental role of calcium dysregulation and its impact on mitochondrial function. Nevertheless, further research is necessary to consolidate the therapeutic opportunities presented in this review.

## 8. Reactive Oxygen Species and Mitochondrial Dysfunction

ROS are chemically reactive molecules derived from oxygen metabolism that play crucial roles in cell signalling and homeostasis. Nevertheless, their excessive production can lead to oxidative stress, causing damage to cellular components, including proteins, lipids, and DNA [204].

Mitochondria, the energy-producing organelles within cells, are a significant source and target of ROS because of aerobic respiration and oxidative phosphorylation (OXPHOS) processes. Hence, recent studies have revealed that mitochondria are primarily responsible for generating significant amounts of ROS, like superoxide anion, oxygen free radicals, hydroxyl radicals, and hydrogen peroxide, through the mitochondrial electron transport chain (ETC) [205]. Consequently, these ROS may exacerbate oxidative stress, leading to cellular damage [206]. Moreover, other cellular sources of ROS include peroxisomes, endoplasmic reticulum, and phagocytic cells [207,208,209]. In this line, ROS can negatively impact mitochondrial function through various mechanisms. Firstly, they can cause oxidative damage to mitochondrial proteins, lipids, and mitochondrial DNA, impairing their function and replication [204,210,211,212]. Secondly, ROS can disturb the electron flow within the ETC, leading to inefficient ATP production and electron leakage, resulting in more ROS generation [213]. Additionally, excessive ROS can disrupt mitochondrial membrane potential [214,215,216], which may have negative effects on cellular signalling pathways. For instance, this increased permeability results in the formation of pores, enabling the transfer of various substances from the mitochondria’s intermembrane space to the cytosol. Within these substances can be found different types of proteins, such as cytochrome c hemoprotein, intermembrane space proteins, and different endonucleases. Then, once these proteins are released into the cytosol, they trigger a cascade of events that activate caspase signalling. Caspases are a family of proteins that play a critical role in apoptosis, promoting cell death and impacting tissue integrity and function [217,218].

To counteract ROS-induced damage, cells have evolved an intricate antioxidant defense system. This system comprises antioxidant enzymes, such as superoxide dismutase, catalase, and glutathione peroxidase, which scavenge and neutralise ROS. Additionally, non-enzymatic antioxidants, including vitamin C, vitamin E, and glutathione, play essential roles in maintaining cellular redox balance [219,220,221,222,223,224]. In this line, mitochondrial dysfunction, which implies an inability to overcome this oxidative stress, has been implicated in various diseases, including neurodegenerative disorders. Hence, different neurodegenerative disorders have been highlighted by the previous literature as being characterised by mitochondrial dysfunction and oxidative stress [225]. Thus, recent researchers confirm the idea that alterations in brain metabolism, linked to neuro-inflammation and mitochondrial dysfunction, which lead to oxidative stress, significantly contribute to the underlying mechanisms of mild cognitive impairment, Parkinson’s disease, and Alzheimer’s disease [226,227].

In Alzheimer’s disease, specific histopathological features characterise brain tissue. These hallmarks include intracellular neurofibrillary tangles and extracellular senile plaques composed of the amyloid-beta peptide in an aggregated form, often associated with metal ions such as copper, iron, or zinc [228,229,230,231,232]. These metal ions can play a role in the generation of ROS when bound to the amyloid-beta peptide. When redox-active metal ions like copper or zinc bind to the amyloid-beta peptide, they facilitate the production of ROS [233]. The most reactive ROS produced in this process is the hydroxyl radical. Consequently, it may cause oxidative damage not only to the amyloid-beta peptide itself but also to surrounding molecules, including proteins, lipids, and other cellular components [234]. For example, proteins crucial for glycolysis and ATP production may become dysfunctional due to this oxidative stress. This disruption in brain energy metabolism, resulting from impaired glycolysis and ATP production, appears to be a critical event in Alzheimer’s disease [235]. Conversely, reduced ATP levels could lead to electron leakage and an upsurge in mitochondrial production of ROS, constituting an additional source of oxidative stress in Alzheimer’s disease [236]. Moreover, oxidative stress has been pointed out by its implication in the clearance of amyloid-beta peptide since it was hypothesised that amyloid-beta peptide could oxidise low-density lipoprotein receptor-related protein (LRP1). LRP1 is a multifunctional protein responsible for various functions, including the efflux of amyloid-beta peptide from the brain to the blood through the blood–brain barrier [237,238]. Moreover, when LRP1 is affected by oxidative stress and its function is impaired, it could disrupt the normal clearance mechanism of amyloid-beta peptide from the brain, potentially contributing to its accumulation having detrimental effects in Alzheimer’s disease, as several studies pointed out, where it was suggested that LRP1 activity is diminished in AD [239]. Finally, oxidative stress may also affect the protein tau, which is thought to contribute to the formation of neurofibrillary tangles. Hence, 4-hydroxynonenal (4-HNE), a lipid peroxidation product, has been proposed because of its capability to modify the conformation of protein tau, favouring neurofibrillary tangle formation [240]. In addition, it has been proposed that a deficiency in antioxidant systems, such as catalase activity, may be related to the onset of AD. Hence, it has been highlighted how amyloid-beta peptide may be responsible for hydrogen peroxide accumulation, enhancing oxidative stress, and compromising AD, probably explained through the fact that amyloid-beta peptide could bind to catalase, diminishing its activity, which promotes an oxidative environment [241].

Regarding PD, it is well known how its pathophysiology involves a reduction of dopamine due to the impairment of dopamine-producing neurons in the substantia nigra [242]. Nevertheless, it has been proposed that another protein, alpha-synuclein, may also be associated with the pathophysiology of Parkinson’s disease. In this line, the previous literature suggested how mutations in a gene responsible for alpha synuclein production led to the formation of a mutant protein, which has the ability to induce the accumulation of dopamine in the cytoplasm of neurons [243,244]. Moreover, this mutant alpha-synuclein disrupts the integrity of the vesicles that contain dopamine, resulting in their permeation and subsequent leakage of dopamine into the cytoplasm. Therefore, the released dopamine suffers autoxidation, leading to the generation of hydrogen peroxide, superoxide molecules, and toxic dopamine-quinone species, promoting an oxidative environment [245], which can contribute to cellular damage and compromise Parkinson’s development. Furthermore, previous researchers have demonstrated that mutant alpha synuclein can alter the morphology of mitochondria in neurons of the central nervous system in vivo in an age-dependent manner, and in vitro experiments have revealed that this mutant form also disrupts mitochondrial transport [246]. These findings suggest that the presence of this alpha-synuclein mutation may have significant effects on mitochondrial dynamics and function, potentially contributing to the pathogenesis of PD. Finally, regarding the expression of antioxidant substances that may reduce oxidative surroundings, mutant alpha-synuclein has also been proposed because of its capability of decreasing the expression and activity of different enzymes, such as catalase [247].

## 9. Inflammation and Mitochondrial Impairment in Brain Disease

**Figure 3:**
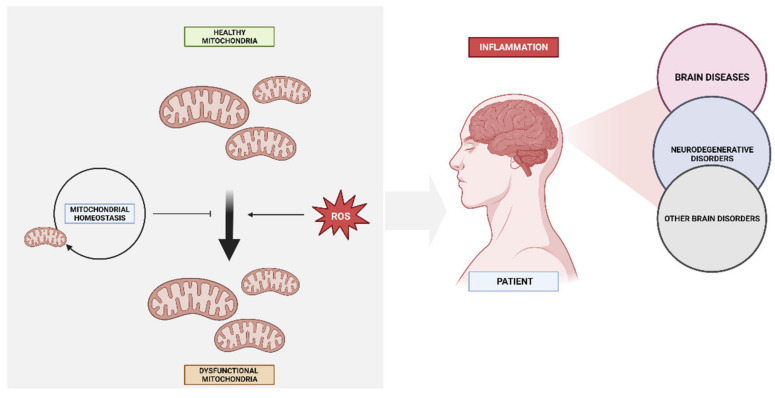
Brain diseases brought on by inflammation and resulting from mitochondrial malfunction.

The human brain represents the pinnacle of evolutionary progress due to its intricate functionality, enabling cognitive processes, memory retention, motor control, and emotional experiences. The primary objective of maintaining a healthy lifestyle is to ensure optimal cognitive function of the brain throughout the entirety of one’s lifespan. The incidence of neurological disorders and the challenges associated with maintaining optimal brain health escalate concomitantly with the advancing age of the populace. There have been numerous neurological conditions that have been associated with compromised cerebral function and adverse health consequences in individuals [248]. There are three main categories to categorise neurological diseases that lead to cognitive impairment (Figure 3). Firstly, brain diseases that cause obvious damage to brain structures include stroke, head trauma, malignant brain tumours, meningitis, and a variety of communication and sensory abnormalities. Brain disorders (such as PD and AD) and mental disorders (such as schizophrenia, depression, bipolar disorder, alcoholism, and drug misuse) are examples of functional brain disorders with a demonstrable breakdown of brain connections or networks. Finally, migraines, sleep difficulties, and other neurological conditions that do not cause obvious damage to the brain [248]. Mitochondrial dysfunction can be both the cause and consequence of inflammatory processes and lead to metabolic adaptations that can be either protective or progressively detrimental to our brains and lead to any of the above processes and diseases [249].

Stroke is a major contributor to adult mortality and disability rates. Neuronal mortality caused by ischemia and reperfusion (I/R) has been linked to mitochondrial malfunction. The survival of neurons and their functional enhancement depend on mitochondria [250]. Yan et al. specified that the mitochondrial disease that contributes to neuronal mortality following stroke includes calcium overload, opening of the mitochondrial permeability transition pore (mPTP), and excessive formation of ROS [251]. In this regard, Monsour et al. pointed out that restoring mitochondrial integrity may be at the root of the therapeutic benefits associated with stem cell therapy, which has the potential to revolutionise stroke treatment thanks to recent advances in cell-based therapies. Reducing oxidative damage and neuroinflammation after stroke and reperfusion injury depends critically on properly functioning mitochondria [252]. Additionally, the most prevalent type of malignant brain tumour is called glioblastoma (GMB). There is mounting evidence that mitochondrial dysfunction is crucial to the development of GBM. As mentioned, mitochondria play a crucial role in controlling cellular metabolism, oxidative stress, and apoptosis. Tumour-associated macrophages and microglia (TAM) are a heterogeneous population of myeloid cells that, in general, provide an immunosuppressive milieu to support tumour growth and dominate the immune microenvironment in GBM [253].

To further enhance our comprehension of mitochondrial dysfunction and its correlation with brain disease, it is imperative to delve into the realm of sepsis-associated encephalopathy (SAE). This neurological complication, which arises frequently in conjunction with sepsis, remains insufficiently comprehended. Notably, SAE is often accompanied by cerebral coagulopathy, ischemia, and brain inflammation [254]. From a medical perspective, it is important to note that SAE (Severe Acute Encephalopathy) is commonly linked to a significant risk of mortality. Additionally, individuals who do survive this condition often experience persistent autonomic nerve dysfunction, delirium, or cognitive dysfunction to varying extents. In recent times, there has been a growing emphasis on the concept of “cytopathic hypoxia”, which refers to the disturbance of cellular bioenergetics in the context of sepsis. This phenomenon has shed light on the profound consequences of mitochondrial damage, leading to the failure of vital organs and ultimately resulting in mortality [255]. As previously stated, this condition of mitochondrial dysfunction is correlated with impaired blood–brain barrier function in various neurological disorders, including stroke. Despite the observations, the examination of modified mitochondrial dynamics (specifically fission, fusion, and mitophagy) in relation to blood–brain barrier (BBB) impairment and consequent septic encephalopathy has not been sufficiently explored [256,257].

Focusing this review on other consequences of mitochondrial malfunction and inflammation processes, much of the literature focuses on neurodegenerative diseases, as mentioned in previous sections. Related to this, the antioxidant capacity of a cell decreases as a result of oxidative stress caused by cellular damage. Therefore, maintaining cellular homeostasis requires precise regulation of ROS generation. In fact, mitochondria in the brain are widely believed to mediate allostasis, the ability to respond to stress via a complex interaction between the autonomic, metabolic, and immune systems to preserve cellular homeostasis [258]. Mitochondrial constituents are displaced into intracellular or extracellular compartments in response to stimuli such as oxidative stress and deficient quality control. Using metabolic stress mediators like glucocorticoids and catecholamines, mitochondria function as highly dynamic integrators in this process, allowing cells to respond to stresses in adaptive ways [259]. By upsetting cellular homeostasis, which is created by chronic stress, it is thought to put a person at risk for neurodegenerative disease. This process is characterised by alterations in mitochondrial function and structure that ultimately lead to oxidative stress, inflammation, mitochondrial DNA damage, and cell death [260,261]. In this regard, proinflammatory mediators, such as cytokines and chemokines, are synthesised and released during neuroinflammation, a shared feature of neurodegenerative disorders [261]. However, the mechanisms that cause mtDNA unloading are mostly unclear. Freely moving mtDNA can be transported into the extracellular compartment through either the complete ejection of nucleoids or the formation and release of extracellular vesicles. Discarded mtDNA might act as a damage-associated molecular pattern (DAMP) by attaching to danger signal receptors, eliciting an inflammatory response from the innate immune system. Mitochondrial DAMPs may have a role in the development of a wide range of neurological illnesses, all of which are associated with neuroinflammation [262]. Bipolar disorder, multiple sclerosis, PD, schizophrenia, melancholy, autism, and chronic fatigue syndrome are examples of such conditions.

Multiple sclerosis (MS) has been studied extensively over the past decade, and numerous studies have shown a link between neuroinflammation and mitochondrial abnormalities [263]. Large numbers of activated microglia and other blood-borne leukocytes (macrophages, T- and B-cells) contribute to tissue harm (demyelination, oligodendrocyte loss, neurodegeneration) in early MS lesions [264]. Similarly, neuronal damage in the later stages of MS is always accompanied by inflammation; however, the magnitude and cellular composition of infiltrates may vary from the first stages of the disease [265]. Additionally, in recent years, it has been observed that some people with autism spectrum disorder (ASD) also suffer from inflammation, mitochondrial malfunction, and/or oxidative stress [266]. Postmortem brain tissues from people with ASD have been shown to have higher levels of oxidative stress than control samples. Oxidative damage to proteins, lipids, and DNA, as well as changes in the activity of enzymes crucial in redox metabolism, have all been shown in these investigations [267].

Rajasekaran et al. also showed that perturbation of mitochondrial network dynamics may lead to inflammatory pathologies in other nervous system disorders. For instance, schizophrenia is characterised by mitochondrial deficiency, altered redox balance, and chronic low-grade inflammation. It is hypothesised that oxidative/nitrosative stress responses resulting from mitochondrial dysfunctions may activate immuno-inflammatory pathways, leading to neuroprogressive alterations in schizophrenia [268]. While the majority of MS patients appear to have widespread mitochondrial dysfunction and impaired ATP production [269], the findings in patients with Parkinson’s disease [270], autism [271], depression [272], bipolar disorder [273], schizophrenia [268], and chronic fatigue syndrome [274] are still completely understood, likely reflecting the fact that these diagnoses do not represent a disease with a unified pathogenesis and pathophysiology. However, research has revealed that the presence of chronic oxidative stress is nearly universal among study cohorts of patients with each diagnosis. This condition is characterised by elevated reactive oxygen and nitrogen species and/or decreased glutathione levels, as well as chronic systemic inflammation with elevated pro-inflammatory cytokine levels [275].

The modulation of mitochondria, either directly or indirectly via bioenergetic pathways, may be effective in reducing metabolic dysfunction and neuroinflammation in brain disease patients [276]. Therefore, it is imperative to recognise the significance of mitochondrial inflammation as a crucial diagnostic focus and advocate for further investigation to explore its potential as a therapeutic target for the management of conditions such as AD, multiple sclerosis, MS, or PD. The transplantation of mitochondria holds promise as a potential method for alleviating harm to brains afflicted with disease or injury, and further investigation is warranted to enhance the techniques used for delivery [111,277]. The efficient treatment of neurodegenerative disorders (NDs) continues to be impeded by the formidable blood–brain barrier (BBB). Considering the numerous accomplishments demonstrated through surgical interventions and highly invasive methodologies, their clinical implementation remains restricted due to various concerns surrounding the enduring advantages, attributable to the potential compromise of the blood–brain barrier. Nanotherapeutics possessing the potential to traverse the blood–brain barrier (BBB) without causing harm to the barrier have been postulated and demonstrated in various instances as a viable alternative for the purpose of decelerating or reversing neurodegeneration [278].

### Inflammation in Metabolic Diseases: A Progress to Brain Diseases

Metabolic disorders have been observed to potentially correlate with a decline in cognitive functions, including but not limited to memory, abstract reasoning, verbal fluency, attention, and psychomotor speed [279]. Obesity has the potential to induce alterations in the hippocampus, a prominent anatomical region crucially implicated in the processes of learning and memory. Based on the reviewed evidence, it appears that obesity, through its inflammatory effects, can result in the impairment of brain structures like the frontal cortex and corpus callosum, which are crucial for plasticity processes and cognitive functions. Furthermore, it has been observed that obesity is correlated with deviations in the white matter and glial cells within the brain, potentially leading to a decline in cognitive processing velocity [280]. There may exist a potential non-linear correlation between lipids and cognitive function, which could be influenced by age and the pro-inflammatory process. Disturbances in carbohydrate metabolism may give rise to cognitive impairment, encompassing deficits in memory, verbal fluency, and abstract reasoning. Obesity and metabolic disorder studies, both preclinical and clinical, have revealed brain mitochondrial dysfunction. Since neuronal cells have a high energy requirement and mitochondria play a crucial role in sustaining a constant energy supply, alterations in mitochondrial activity result in neuronal damage and dysfunction, and therefore neurotoxicity [281,282].

Specifically, recent evidence indicates a compelling association between metabolic disorders and AD. In the past decade, there has been significant emphasis placed on elucidating the intricate relationship between lipid metabolism dysfunction and AD. In this discourse, we shall delve into the various facets of lipid regulation, encompassing alterations in cholesterol levels, the role of apolipoproteins and leptin in bodily functions, and the interconnection of these factors with the pathogenesis of Alzheimer’s disease. Despite the extensive body of literature at our disposal, there remains a need for further elucidation and clarification of numerous aspects. However, the pathway has already been established to directly link components of lipid regulation to AD [283].

Additionally, there is an increasing body of evidence that has established a strong association between metabolic disease and the occurrence of neurovascular disorders as well as cognitive decline. In our study utilising a murine model, we have replicated the effects of a high-fat, high-sugar diet, which simulates the development of type 2 diabetes mellitus (T2DM) in humans [284]. Our findings demonstrate that the presence of pro-inflammatory agents and changes in immune responses lead to detrimental effects on the structure of the blood–brain barrier (BBB). Consequently, this disruption triggers a metabolic phenotype characterised by inflammation [284]. Moreover, the accumulating evidence from the past few decades substantiates the significant involvement of central insulin resistance (IR) associated with obesity and type 2 diabetes (T2D) in the progression of cognitive impairment, which includes its impact on the plasticity of hippocampal synapses. Significantly, insulin resistance (IR) can be effectively addressed by optimising lifestyle factors **[285]**. These factors encompass engaging in regular physical activity while avoiding a sedentary lifestyle, adopting an improved diet with a focus on increased fibre intake, and ensuring sufficient sleep **[286]**. The condition known as insulin resistance (IR) is situated at the crucial intersection where obesity intersects with both metabolic and cognitive dysfunction. Considering the significance of insulin resistance (IR) in the development of numerous chronic diseases prevalent in the 21st century as well as its potential for reversal, it is imperative that we collectively adopt and promote enhanced lifestyles to enhance the future health and well-being of the population. In this regard, Shannon et al.’s findings elucidate a groundbreaking mechanism of action of pioglitazone that is pertinent to the pathogenesis of type 2 diabetes mellitus These findings propose that directing attention towards pyruvate metabolism could potentially pave the way for the creation of innovative and efficacious therapies for T2DM [287].

## 10. Mitochondria and Neurotransmitter Systems in Psychiatric Illnesses

Mitochondria are essential organelles responsible for energy production and cellular homeostasis. Disruptions in mitochondrial function have been implicated in various neurodegenerative diseases, and recent studies have begun to uncover their involvement in psychiatric illnesses as well. Mitochondrial dysfunction in the central nervous system can lead to increased oxidative stress, impaired energy production, and dysregulation of ion homeostasis, which may contribute to neuronal damage and functional deficits seen in psychiatric disorders [288].

In this line, several psychiatric illnesses such as depression, Parkinson’s disease, Alzheimer’s disease, schizophrenia, bipolar disorder, and anxiety disorders have been related to alterations in serotonin, dopamine, glutamate, and gamma-aminobutyric acid (GABA) signalling [242,289,290,291,292,293]. Hence, it has been described in the recent literature how dysregulated neurotransmitter signalling may lead to increased oxidative stress and mitochondrial damage, further exacerbating the dysfunction of these organelles [294,295]. Conversely, mitochondrial dysfunction can disrupt neurotransmitter release and uptake, causing imbalances in neuronal communication and synaptic plasticity [296].

Firstly, it has been largely described how, in depression, there may be an imbalance in the production and release of neurotransmitters such as dopamine, serotonin, glutamate, or noradrenaline. These essential chemical messengers play a crucial role in regulating mood, cognition, and various physiological processes, and any disruption in their delicate equilibrium can significantly impact mental health and cognitive function, contributing to the onset and progression of these debilitating conditions [297]. Moreover, recent literature has pointed out how mitochondrion dysfunction, including lower respiratory chain enzyme activity and decreased ATP production, may be related to higher oxidative stress and, consequently, be associated with major depression. Similar results were found regarding higher production of ROS species and modifications in oxidative stress enzyme activities [298]. In this line, it is important to point out modifications that could affect enzyme activities, as they may modulate neurotransmitter levels. This is the case with monoamine oxidase activity. Oxidative deamination of monoamine neurotransmitters and serotonin metabolism is carried out through the monoamine oxidase (MAO) enzyme. Nevertheless, this process could enhance oxidative subproducts, such as hydrogen peroxide, leading to an overproduction of ROS. Excessive ROS production can result in detrimental effects on neurons, leading to apoptosis and mitochondrial dysfunction [299]. Thus, raised MAO levels were found in patients with depression, suggesting how this MAO activity may compromise depression management [300]. Additionally, 2,3-dioxygenase (IDO) is another key enzyme that takes part in neurotransmitter metabolism. It mediates tryptophan catabolites synthesis to obtain tryptophan as a precursor of serotonin. Thus, the prior literature suggested that the increased activity of IDO shows a negative correlation with the concentrations of serotonin and tryptophan and a positive correlation with the severity of depression [301]. These results may be explained by the fact that in this metabolic pathway, IDO transforms tryptophan into kynurenine metabolites, such as 3-hydroxy-kynurenine, which has been related to oxidative stress increasing the production of ROS, which may compromise depression status [302].

Regarding Parkinson’s disease, as mentioned above, a mutant form of an enzyme, alpha synuclein, has been identified for its capability of disturbing the integrity of the vesicles that contain dopamine, promoting their breakup, and favouring the release of dopamine into the cytoplasm. As a result, an autoxidation process occurs, promoting ROS production and cellular harm [303]. Moreover, recent literature suggests that oxidative stress can further exacerbate neurotransmitter dysregulation in Parkinson’s disease. ROS can impair the synthesis, release, and reuptake of neurotransmitters, disrupting their normal function and exacerbating neuronal damage [304]. Additionally, the loss of dopamine-producing neurons may increase oxidative stress due to the decreased ability to produce antioxidant enzymes, creating a vicious cycle of oxidative damage and neurotransmitter dysregulation [305]. Consequently, the loss of dopamine-producing neurons in the substantia nigra disrupts the delicate balance of neurotransmitters in the brain. Dopamine plays a crucial role in regulating movement, mood, and cognition. As dopamine levels decline, other neurotransmitter systems, such as glutamate and acetylcholine, become dysregulated, contributing to the motor and non-motor symptoms seen in Parkinson’s disease [306,307].

Concerning Alzheimer’s disease, two neurotransmitters are relevant: acetylcholine and glutamate. Hence, raised glutamate and decreased acetylcholine levels were found in Alzheimer’s patients [308]. As the primary excitatory transmitter of the brain, glutamate plays a pivotal role in all cognitive functions, serving as the main transmitter of cortical and hippocampal pyramidal neurons. Additionally, glutamate and its receptors are instrumental in long-term potentiation, a critical process believed to be the foundation for learning and memory [309]. Moreover, it has been found in Alzheimer’s patients that there is the presence of malondyaldehyde and 4-hydroxynonenal, which are subproducts of lipid peroxidation, because of ROS activity. It has been described by previous authors how they may affect glutamate transporter activity, compromising Alzheimer’s disease [310]. Regarding acetylcholine, it has been found that Alzheimer’s patients present a decreased presence of this neurotransmitter. It may be explained due to the activity of ROS in acetylcholine levels, as it was reported in the previous literature that the rise of ROS formation could promote acetylcholinesterase activity [311], which consequently may reduce acetylcholine levels. Furthermore, the decline in acetylcholine levels has been linked to memory impairment and cognitive deficits in Alzheimer’s patients [312].

Regarding anxiety disorders, recent studies conducted over the past few years have provided strong evidence suggesting that anxiety disorders may exhibit decreased antioxidant defences and an elevation in oxidative damage to essential biomolecules such as proteins, lipids, and nucleic acids. Thus, heightened ROS activity may contribute to cellular damage, including the oxidation of several structures such as membrane phospholipids, proteins, as well as nuclear and mitochondrial DNA, causing lipid peroxidation and protein dysfunction [313]. Particularly, oxidative modifications to proteins have been suggested as a key contributor to the development and advancement of various psychiatric disorders, including anxiety and depressive disorders. For example, it has been described how the impairment of the Lon protease in mitochondria (essential for preserving mitochondrial integrity by selectively eliminating oxidised intra-mitochondrial proteins) has been linked to various neurological disorders [314,315]. Additionally, recent literature revealed a potential link between PSMD9, a protein subunit of the 26S proteasome complex, and generalised anxiety disorder [316]. Proteasomes play a critical role as essential proteolytic enzymes in eukaryotes, safeguarding proteostasis in the cell’s cytoplasm, nucleus, and endoplasmic reticulum [317]. Hence, failure of proteasomes or other components within the ubiquitin-proteasome system might be associated with impaired clearance of defective proteins in the cytoplasm, nucleus, and endoplasmic reticulum, promoting protein aggregation, cytotoxicity, and cellular damage [318,319]. Thus, these findings suggest that reduced proteasome activity could potentially increase negative oxidative effects, further exacerbating the adverse outcomes of anxiety disorders. Moreover, regarding GABA (γ-aminobutyric acid) modulation, several authors found a relationship between ROS and its regulation [320]. GABA is a neurotransmitter that plays an essential role in neuronal inhibition. GABA A receptor activation enhances membrane permeability to chloride and bicarbonate ions, resulting in an overall influx of anions that typically hyperpolarizes the cells, thereby reducing cellular excitability [321]. Hence, it has been described how ROS may disturb GABA A receptor subtypes since it may present potentiating or inhibiting actions on them [322]. Thus, anxiety disorders may be affected since the aim of these disorders is to re-establish hyperpolarization in order to reduce neuroexcitability.

## 11. Mitochondrial Biomarkers in Brain Disease

### 11.1. Mitochondrial Biomarkers in Neurodegenerative Disorders

The search for mitochondrial biomarkers in brain disease has predominantly focused on neurodegenerative disorders such as Alzheimer’s disease, Parkinson’s disease, and Huntington’s disease. These disorders are characterised by the accumulation of abnormal protein aggregates, mitochondrial dysfunction, and progressive neurodegeneration.

In this line, neurodegenerative disorders, such as Alzheimer’s disease, Parkinson’s disease, and Huntington’s disease, are characterised by progressive neuronal loss and cognitive decline. Mounting evidence suggests that mitochondrial dysfunction plays a critical role in the pathogenesis of these disorders, leading to a heightened interest in the search for mitochondrial biomarkers [323].

In Alzheimer’s disease, the measurement of mitochondrial biomarkers in cerebrospinal fluid (CSF), such as levels of amyloid-beta, tau, and phosphorylated tau proteins, has shown promise in aiding early diagnosis and tracking disease progression. Increased CSF levels of amyloid-beta and tau proteins, which are involved in the formation of plaques and neurofibrillary tangles in AD, correlate with mitochondrial dysfunction and neuronal damage. Furthermore, decreased levels of mitochondrial enzymes, such as cytochrome c oxidase, have been observed in postmortem brain tissue of Alzheimer’s patients, suggesting impaired oxidative phosphorylation and mitochondrial dysfunction. These findings highlight the potential of CSF biomarkers to reflect mitochondrial alterations and disease severity in Alzheimer’s disease [324].

Neuroimaging techniques have also been employed to assess mitochondrial function in neurodegenerative disorders. Positron emission tomography (PET) imaging using radiotracers specific to mitochondrial function, such as [^18F]fluorodeoxyglucose (FDG), can measure glucose metabolism, which is closely linked to mitochondrial activity. Reduced glucose metabolism in specific brain regions affected by neurodegeneration can serve as an indirect marker of mitochondrial dysfunction. Similarly, magnetic resonance spectroscopy (MRS) can measure brain metabolites associated with mitochondrial function, such as lactate and N-acetylaspartate (NAA), providing insights into cellular energy metabolism and mitochondrial health [325].

In Parkinson’s disease, mitochondrial dysfunction is a well-established feature, particularly in dopaminergic neurons of the substantia nigra. Mitochondrial DNA (mtDNA) mutations and deletions have been identified in the postmortem brains of Parkinson’s patients, suggesting a role for mtDNA as a potential biomarker. Additionally, peripheral levels of mtDNA and markers of oxidative stress, such as 8-hydroxy-2’-deoxyguanosine (8-OHdG), have been associated with disease severity and progression. These peripheral biomarkers offer non-invasive options for monitoring mitochondrial dysfunction in Parkinson’s disease [326].

Neuroimaging techniques, such as PET and single-photon emission computed tomography (SPECT), have also been used to evaluate mitochondrial function in Parkinson’s disease. PET imaging with radiotracers that target mitochondrial complex I activity, such as [[^18F]N-methyl-2-(4-fluorophenyl)-6-hydroxybenzothiazole (FMT), can provide insights into mitochondrial dysfunction in specific brain regions. SPECT imaging using radiotracers that measure dopamine transporter activity can indirectly reflect mitochondrial function, as dopaminergic neurons rely heavily on mitochondrial energy production. These imaging modalities offer a valuable means of assessing mitochondrial dysfunction in Parkinson’s disease and monitoring disease progression [327].

In Huntington’s disease, mitochondrial dysfunction has been implicated in disease pathology. Peripheral levels of mitochondrial biomarkers, such as mtDNA content and markers of oxidative stress, have been found to correlate with disease severity and progression. Furthermore, neuroimaging techniques, including PET and MRS, have demonstrated altered glucose metabolism and mitochondrial activity in specific brain regions affected by Huntington’s disease [328].

Moreover, emerging evidence suggests that the analysis of mitochondrial biomarkers in postmortem brain tissue can provide valuable insights into the role of mitochondrial dysfunction in neurodegenerative disorders. Techniques such as immunohistochemistry, electron microscopy, and molecular analyses allow researchers to investigate mitochondrial structure, distribution, and molecular alterations in specific brain regions affected by the disease. Abnormalities in mitochondrial morphology, such as fragmented or swollen mitochondria, along with changes in mitochondrial protein expression, oxidative damage, and DNA mutations, have been observed in neurodegenerative disorders [329].

In recent years, there has been a growing interest in the use of liquid biopsy techniques to detect mitochondrial biomarkers in neurodegenerative diseases. Liquid biopsy involves the analysis of biological fluids such as blood, urine, and cerebrospinal fluid (CSF) to identify biomarkers associated with disease. The analysis of circulating mitochondrial DNA (mtDNA) and mitochondrial-derived peptides (MDPs) in plasma or serum has shown promise as potential non-invasive biomarkers for neurodegenerative disorders. These biomarkers can be obtained through minimally invasive procedures, offering a convenient and accessible method for disease monitoring and early detection [330].

Furthermore, the investigation of mitochondrial biomarkers in the context of therapeutic interventions is gaining attention. As researchers explore potential therapies targeting mitochondrial dysfunction, the assessment of mitochondrial biomarkers can serve as valuable tools for evaluating treatment efficacy and monitoring disease progression. For example, in clinical trials investigating mitochondria-targeted therapies, such as antioxidants or mitochondrial protective agents, the measurement of mitochondrial biomarkers can provide insights into the impact of the intervention on mitochondrial function and the overall course of the disease [331].

Although promising, several challenges and limitations exist in the field of mitochondrial biomarkers. Variability in sample collection, storage, and processing techniques can influence biomarker measurements and introduce inconsistencies across studies. Standardisation of protocols and the establishment of reference ranges for biomarker levels are essential for the widespread use and interpretation of mitochondrial biomarkers. Additionally, the heterogeneity of neurodegenerative disorders poses challenges in identifying specific biomarkers that can differentiate between different disease subtypes and stages [332].

In conclusion, the analysis of mitochondrial biomarkers in neurodegenerative disorders offers promising avenues for understanding the role of mitochondrial dysfunction in disease pathogenesis, early diagnosis, and monitoring treatment responses. Utilising a multidimensional approach that combines CSF biomarkers, peripheral blood biomarkers, miRNAs, metabolomics, advanced imaging techniques, and postmortem brain analysis, researchers aim to refine and validate these biomarkers. The continued advancements in mitochondrial research and the identification of reliable biomarkers will contribute to improved disease management, personalised treatments, and the development of novel therapeutic interventions targeting mitochondrial dysfunction.

### 11.2. Mitochondrial Biomarkers in Psychiatric Illnesses

Psychiatric illnesses, including major depressive disorder, bipolar disorder, and schizophrenia, are complex and multifactorial conditions that significantly impact the well-being and quality of life of affected individuals. While these disorders have traditionally been associated with neurotransmitter imbalances and abnormalities in brain circuitry, emerging evidence suggests that mitochondrial dysfunction plays a crucial role in their pathogenesis. The identification and characterisation of mitochondrial biomarkers in psychiatric illnesses have garnered considerable attention in recent years, offering new insights into disease mechanisms, facilitating early diagnosis, and guiding personalised treatment approaches [333].

In major depressive disorder, alterations in mitochondrial function have been observed, leading to the exploration of mitochondrial biomarkers as potential diagnostic tools and treatment response predictors. Peripheral levels of mitochondrial DNA (mtDNA) have shown promise as biomarkers for differentiating subtypes of depression, monitoring treatment efficacy, and predicting outcomes. Additionally, markers of oxidative stress and mitochondrial enzyme activity have been investigated, providing further insights into mitochondrial dysfunction in depression [334].

Bipolar disorder, characterised by cyclic mood swings between manic and depressive episodes, has also been associated with mitochondrial dysfunction. Mitochondrial biomarkers, including mtDNA levels and markers of oxidative stress, have demonstrated potential for distinguishing between different mood states, predicting relapse, and monitoring treatment response. These biomarkers offer the possibility of developing targeted interventions that directly address mitochondrial dysfunction in bipolar disorder.

Schizophrenia, a complex psychiatric disorder, is characterised by disturbances in perception, cognition, and emotional regulation. Growing evidence suggests that mitochondrial dysfunction contributes to the pathogenesis of schizophrenia, and mitochondrial biomarkers have been investigated to improve our understanding of the disorder. Peripheral levels of mtDNA, markers of oxidative stress, and mitochondrial enzyme activity have been explored as potential diagnostic markers and indicators of disease severity. These biomarkers may help identify specific subgroups of patients with distinct mitochondrial profiles, facilitating the development of tailored treatment strategies [293].

The identification and validation of reliable mitochondrial biomarkers in psychiatric illnesses face several challenges. Standardisation of sample collection and measurement techniques, as well as the establishment of reference ranges and diagnostic thresholds, are crucial for the widespread use and interpretation of these biomarkers. Longitudinal studies with large cohorts are needed to validate their diagnostic and prognostic value, monitor treatment response, and evaluate their potential for guiding personalised interventions. Moreover, the exploration of mitochondrial biomarkers in psychiatric illnesses can shed light on the potential involvement of genetic factors. Mitochondrial DNA (mtDNA) mutations and deletions have been identified in individuals with schizophrenia and bipolar disorder, suggesting a role for mitochondrial genetic variants in these conditions. Investigating mtDNA biomarkers in peripheral blood samples may provide valuable insights into the contribution of mitochondrial genetics to the development and progression of psychiatric illnesses [335].

In recent years, advancements in neuroimaging techniques have allowed for the non-invasive assessment of mitochondrial function in psychiatric illnesses. Positron emission tomography (PET) imaging using radiotracers targeting mitochondrial activity has provided valuable information about regional brain metabolism and mitochondrial function in individuals with psychiatric disorders. This imaging approach has revealed abnormalities in specific brain regions associated with mood regulation, cognitive processing, and emotion processing, further supporting the involvement of mitochondrial dysfunction in psychiatric pathophysiology [336].

Furthermore, the integration of mitochondrial biomarkers with other clinical measures, such as cognitive assessments, symptom severity scales, and treatment response monitoring, can enhance the precision and efficacy of personalised treatment strategies. By identifying biomarkers that correlate with specific symptom clusters or treatment outcomes, clinicians can make more informed decisions regarding medication selection, dosage adjustments, and therapeutic interventions tailored to individual patients [337].

Despite the significant progress made in understanding the role of mitochondrial dysfunction in psychiatric illnesses, several challenges remain. The heterogeneity of these disorders, both in terms of symptom presentation and underlying pathophysiology, poses a challenge in identifying universal biomarkers that are applicable across all individuals with psychiatric illnesses. Large-scale collaborative studies, utilising multiomics approaches and incorporating diverse patient populations, are needed to overcome these challenges and identify robust mitochondrial biomarkers [338].

In conclusion, the investigation of mitochondrial biomarkers in psychiatric illnesses holds promise for advancing our understanding of disease mechanisms, improving early detection and diagnosis, and guiding personalised treatment approaches. Moreover, recent studies have highlighted the involvement of mitochondrial dynamics in psychiatric illnesses. Mitochondrial fusion and fission, processes that regulate mitochondrial morphology and distribution, have been found to be dysregulated in major depressive disorder and bipolar disorder. Altered expression and activity of proteins involved in mitochondrial dynamics, such as dynamin-related protein 1 (Drp1) and mitofusins, have been observed in postmortem brain tissue of individuals with these psychiatric conditions. These findings suggest that disruptions in mitochondrial dynamics contribute to the pathophysiology of these disorders and may represent potential therapeutic targets [339].

Additionally, the investigation of mitochondrial bioenergetics has provided further insights into the metabolic alterations associated with psychiatric illnesses. Studies have demonstrated aberrant mitochondrial respiration and ATP production in individuals with major depressive disorder and bipolar disorder. Dysfunctional mitochondria may contribute to reduced energy availability in affected brain regions, impacting neuronal function and overall mood regulation [340]. Understanding the metabolic changes associated with mitochondrial dysfunction in psychiatric illnesses may lead to the development of novel therapeutic strategies targeting energy metabolism and mitochondrial bioenergetics.

Furthermore, alterations in mitochondrial oxidative stress have been implicated in the pathogenesis of psychiatric illnesses. Mitochondria are major sources of ROS production, and an imbalance between ROS generation and antioxidant defence mechanisms can lead to oxidative stress and cellular damage. Studies have shown increased oxidative stress markers, such as lipid peroxidation products and protein carbonylation, in individuals with major depressive disorder, bipolar disorder, and schizophrenia. Therapeutic interventions aimed at reducing mitochondrial oxidative stress, such as antioxidant supplementation and modulators of antioxidant enzyme activity, may hold potential for managing psychiatric symptoms and improving treatment outcomes [341].

### 11.3. Challenges and Perspectives of Biomarkers in Neurodegenerative Disorders and Psychiatric Illnesses

#### 11.3.1. Challenges

Standardisation: One of the major challenges in the field of mitochondrial biomarkers is the standardisation of sample collection, processing, and measurement techniques. Consistency in methodologies is crucial to ensure the reproducibility and comparability of results across different studies and clinical settings [342].

Reference Ranges and Diagnostic Thresholds: Establishing reference ranges and diagnostic thresholds for mitochondrial biomarkers is essential for their clinical utility. Determining cutoff values that differentiate between healthy individuals and those with psychiatric illnesses requires large-scale studies and validation in diverse populations [343].

Heterogeneity of Psychiatric Illnesses: Psychiatric illnesses exhibit significant heterogeneity in terms of symptom presentation, underlying pathophysiology, and treatment response. Identifying universal mitochondrial biomarkers that are applicable across all individuals with psychiatric illnesses poses a challenge due to this heterogeneity.

Longitudinal Studies and Large Cohorts: Longitudinal studies with large cohorts are needed to validate the diagnostic, prognostic, and treatment monitoring value of mitochondrial biomarkers. These studies require substantial resources and long-term follow-up, but they are crucial for establishing the clinical utility of these biomarkers [344].

#### 11.3.2. Future Perspectives

Multiomics Approaches: Integrating mitochondrial biomarker analysis with genomic, proteomic, and metabolomic data can provide a more comprehensive understanding of the molecular pathways and networks involved in mitochondrial dysfunction in psychiatric illnesses. This integrative approach may lead to the discovery of novel biomarker signatures and therapeutic targets [345].

Personalised Medicine: Mitochondrial biomarkers have the potential to guide personalised treatment approaches in psychiatric illnesses. By identifying biomarkers that correlate with specific symptom clusters or treatment outcomes, clinicians can make more informed decisions regarding medication selection, dosage adjustments, and therapeutic interventions tailored to individual patients [346].

Novel Therapeutic Strategies: The identification of mitochondrial biomarkers opens up avenues for exploring novel therapeutic strategies targeting mitochondrial dysfunction. This includes the use of mitochondrial protective agents, metabolic modulators, and gene therapy approaches aimed at restoring mitochondrial function and improving energy metabolism [347].

Technology Advancements: Continued advancements in neuroimaging techniques and non-invasive assessment methods may enable more precise and convenient measurement of mitochondrial function in psychiatric illnesses. This can facilitate the integration of mitochondrial biomarkers into routine clinical practice [348].

Collaboration and Data Sharing: Large-scale collaborative efforts and data sharing among researchers and institutions are vital for overcoming the challenges associated with mitochondrial biomarkers. Sharing data and resources can accelerate research progress and enhance the reliability and generalizability of findings [349].

## 12. Therapeutic Strategies: Mitochondrial Protective Agents

Mitochondrial damage refers to the alteration or dysfunction of mitochondria, the intracellular structures responsible for producing energy in the form of ATP. Mitochondria are vital for cellular functioning and play a key role in many biological processes. Some mechanisms are involved in mitochondrial damage. One of them is oxidative stress, associated with an imbalance between reactive oxygen species (ROS) production and elimination damaging mitochondrial structures and compromising their function [350]. Oxidative stress is associated with neurodegenerative diseases such as Alzheimer’s and Parkinson’s [47], as well as cardiovascular diseases, diabetes, and cancer [351,352,353]. In addition, Electron transport chain dysfunction leads to inadequate ATP production and increased ROS release, further damaging mitochondria [354]. Moreover, accumulated mitochondrial DNA damage can negatively affect mitochondrial function and contribute to ageing and various age-related diseases, such as cardiovascular diseases and neurodegenerative disorders [355]. Additionally, mitochondrial damage can release pro-apoptotic proteins, thus dysfunctional apoptosis that can contribute to autoimmune diseases, cancer, and neurodegenerative disorders [356]. Finally, imbalances in calcium transport can lead to its accumulation within mitochondria, causing damage and activating apoptosis pathways, promoting cellular dysfunction [197]. This has been implicated in neurological disorders such as Huntington’s disease and amyotrophic lateral sclerosis (ALS) [357,358].

A number of pharmaceutical medications and naturally occurring substances, which have been classified as mitochondrial protective agents, have demonstrated promise in the preservation of mitochondrial integrity and functionality. Mitochondrial damage-associated disorders may potentially benefit from the use of these interventions for treatment or prevention purposes. Previous research has examined the antioxidant and anti-inflammatory characteristics of some substances, such as coenzyme Q10 [359], alpha-lipoic acid [360], melatonin [361], resveratrol [362], carnitine [363], curcumin [364] or metformin [365] as products against mitochondrial dysfunction.

Regarding the evidence for these mitochondrial protective agents, preclinical studies have shown that coenzyme Q10 supplementation improves mitochondrial function, reduces oxidative stress, and provides neuroprotective effects in animal models of neurodegenerative diseases [366,367,368]. Coenzyme Q10 has antioxidant and anti-inflammatory effects and plays a role in energy production and mitochondrial stabilisation, which are mechanisms by which coenzyme Q10 exerts its neuroprotective effects [369]. Conversely, clinical trials have evaluated coenzyme Q10‘s potential benefits in neurodegenerative conditions, such as Parkinson’s and Alzheimer’s disease, reporting controversial findings, with some trials showing promising effects on slowing disease progression [370,371,372] and others finding limited benefits [373,374].

Similarly, for the effects of alpha-lipoic acid in neurological disorders, preclinical studies have described the anti-inflammatory activity of this compound as well as the prevention of neuronal damage caused by reactive oxygen species imbalance, a pathophysiological alteration observed in neurodegenerative diseases [375,376]. In the same way, the effectiveness of resveratrol has been demonstrated in both preclinical and clinical studies.

Resveratrol is recognised for its antioxidant, anti-inflammatory, anti-apoptotic, and anticancer properties. It has been shown to influence mitochondrial function, redox biology, and dynamics in both in vitro and in vivo experimental models. Additionally, resveratrol can mitigate mitochondrial damage caused by specific stressors by enhancing mitochondria-located antioxidant enzymes, thereby reducing reactive species production in these organelles. Furthermore, resveratrol stimulates mitochondrial biogenesis, leading to an improved bioenergetic status of mammalian cells [377].

Likewise, melatonin’s ability to neutralise reactive oxygen species (ROS) and convert them into less harmful species has been clearly described [378]. Its beneficial effects on neurological diseases stem from its potent antioxidant, free radical scavenging, immune system regulation, anti-inflammatory, and circadian rhythm-regulating properties [379]. According to research from experimental animal and clinical studies, melatonin shows promise in the prophylaxis of various neurological diseases, including Alzheimer’s, Parkinson’s, and multiple sclerosis. Although its effects on epilepsy can be both positive and negative, melatonin remains significant for treating other conditions. Clinical studies have explored the usefulness of melatonin for neurological disease treatment, while animal models have demonstrated its effectiveness in reducing neuronal loss and improving cognitive processes.

Regarding the effectiveness of curcumin on neurological diseases, some pharmacological effects have been attributed to it: anticancer, anti-inflammatory, antioxidant, antithrombotic, chemo sensitising and chemo preventive, antiatherosclerosis and cardioprotective, lipid-modifying, antibacterial, antifungal, antiviral, analgesic, pulmonoprotective, antidepressant, and antirheumatic activities [380,381,382,383,384,385]. Furthermore, the safety of curcumin has been established in neurological diseases, but some adverse events (mostly related to gastrointestinal) could be common [386]. Prior research has demonstrated that curcumin exhibits neuroprotective effects attributed to its anti-inflammatory, antioxidant, and anti-protein aggregate activities [387]. Specifically, a previous review [386] showed that curcumin supplementation, either alone or with other ingredients, had beneficial effects for neurological disorders (i.e., migraine, multiple sclerosis, Parkinson’s disease, glioblastoma, and ALS) in terms of inflammation and several associated clinical outcomes. However, curcumin had no beneficial effects in patients with Alzheimer’s disease [386].

A deficiency of L-carnitine can lead to impaired mitochondrial function and cellular metabolic alterations, potentially underlying several disease states [388]. Extensive preclinical and clinical research has confirmed the beneficial role of L-carnitine treatment in conditions such as myalgic encephalomyelitis, chronic fatigue syndrome [389], and neurodegenerative diseases [390]. Recent studies are beginning to unveil L-carnitine’s ability to modulate gene expression and other vital biological processes, in addition to its crucial role in mitochondrial energy metabolism [391,392]. Moreover, both L-carnitine and acetyl-L-carnitine have been linked to the prevention of toxic effects caused by beta-amyloid and the improvement of symptoms in Alzheimer’s disease [388]. These neuroprotective effects may be related to the reduction of amyloid-related mitochondrial dysfunction and a decrease in reactive oxygen species levels [393]. Acetyl-L-carnitine has shown promise in reducing beta-amyloid 1-42-induced protein and lipid oxidation, enhancing antioxidant potential by increasing glutathione and heat shock proteins [394], and preventing ATP depletion induced by beta-amyloid [395]. Experimental studies have demonstrated that treating cortical neurons with acetyl-L-carnitine can counter the neurotoxic effects of beta-amyloid 25–35 fragment [393] and attenuate beta-amyloid 1-42-induced toxicity and apoptosis, which could be attributed to a reduction in protein and lipid oxidation [394]. Hence, the therapeutic efficacy of L-carnitine, which specifically targets mitochondria, is increasingly being comprehended and exhibits significant potential for the treatment of neurological disorders.

Finally, metformin has undergone thorough in vitro and in vivo testing and is an approved compound that targets various pathways, including mitochondrial energy production and insulin signalling. There is increasing evidence for the therapeutic potential of metformin in neurodegenerative diseases; however, in vivo studies have yielded conflicting results. The mechanisms underlying the potential therapeutic effects of metformin in neurodegenerative diseases are linked to its ability to balance survival and death signalling in cells through pathways commonly associated with these conditions [396]. Metformin acts on central metabolism and significant signalling pathways, such as energy sensing (glucose metabolism and AMPK signalling), mTOR signalling, and inflammatory signalling. Additionally, mitochondria play a crucial role in energy production and various other functions essential for central metabolism and cell signalling. Mitochondrial dysfunction is a common feature in all neurodegenerative diseases and underlies β-cell dysfunction in type 2 diabetes mellitus [397]. An essential aspect of mitochondrial dysfunction in neurological diseases is that the demand for tightly controlled energy metabolism in neurons may partially explain some of the vulnerabilities involved in their degeneration. Hence, gaining a deeper understanding of metformin’s mechanisms will aid researchers in the neurodegeneration field to effectively design future studies and trials.

Nevertheless, further well-designed, randomised, placebo-controlled clinical trials are needed to clarify the mechanisms of action, as well as both the clinical efficacy and clinical outcomes, associated with the mitochondrial protective agents used to unequivocally conclude that they are safe and effective compounds for the adjunct treatment of various neurodegenerative/neurological diseases.

## 13. Metabolic Modulators and Mitochondrial Function in Brain Diseases

Mitochondria, the most fascinating cell organelles, are crucial for cellular respiration and energy production. Additionally, they play a vital role in regulating cellular homeostasis by controlling energy production, calcium signalling, cell metabolism, and apoptosis. Mitochondrial dysfunction has been linked to a wide range of diseases, including neurodegenerative disorders, metabolic syndromes, and cardiovascular disease [277]. Mitochondrial dysfunction is a common feature of many diseases, including neurodegenerative disorders. Specifically, glucose metabolism dysregulation reduces energy generation in the brain and negatively impacts neuronal function; lipid metabolism alteration increased oxidative stress and accumulation of waste products; and dysregulation of calcium homeostasis ultimately contributes to disease pathogenesis [398,399].

The aetiology and pathogenesis of some neurodegenerative disorders and brain diseases such as Alzheimer’s, Parkinson’s, and Huntington’s disease exhibit a complex interaction between metabolism and mitochondrial function. Mitochondrial dysfunction and metabolic disorders can be key factors in the pathogenesis and progression of these diseases, highlighting the importance of further research and understanding of this relationship to develop effective therapeutic approaches to address these conditions. Specifically, in Alzheimer’s, there is an abnormal accumulation of beta-amyloid and tau proteins in the brain, leading to the formation of plaques and neurofibrillary tangles [400]. These protein aggregates can impair mitochondrial function and generate oxidative stress, resulting in neuronal damage and reduced energy production in mitochondria [401]. Additionally, alterations in brain glucose metabolism, known as cerebral insulin resistance, have also been linked to mitochondrial dysfunction in Alzheimer’s [402]. Similarly, in Parkinson’s disease, the presence of alpha-synuclein protein aggregates, known as Lewy bodies, has been identified in neurons [403]. These aggregates can interfere with mitochondrial function and energy production in brain cells [404]. Moreover, genetic mutations associated with Parkinson’s directly affect mitochondrial function, suggesting a connection between metabolic disorders and mitochondrial dysfunction in this disease [405]. In the same way, Huntington’s disease is caused by an abnormal expansion of the CAG trinucleotide repeat in the huntingtin gene [406]. This mutation leads to the formation of toxic proteins that accumulate in brain cells, including mitochondria, affecting their function and resulting in decreased ATP production [93]. Mitochondrial dysfunction and energy metabolism alteration are closely linked in Huntington’s disease [407].

Several metabolic modulators have been studied in relation to mitochondrial function in the brain. These compounds have the ability to influence cellular metabolism and mitochondrial activity, which can have beneficial effects on brain function and the management of neurodegenerative diseases [408]. Specifically, the effects of alpha-lipoic acid [409], coenzyme Q10 [410], nicotinamide adenine dinucleotide (NAD+) [411], folic acid (vitamin B9) [412], creatine [413], or resveratrol [414] have been analysed, obtaining some controversial results during the last years. These metabolic modulators show promise in improving mitochondrial function in the brain and could offer therapeutic approaches for the treatment and prevention of brain diseases related to mitochondrial dysfunction. However, it is important to note that research in this field is still ongoing, and more clinical studies are needed to determine their efficacy and safety in the context of neurodegenerative diseases.

Regarding clinical evidence of metabolic modulators, preclinical and clinical studies have investigated their effectiveness in improving mitochondrial function and managing brain diseases. Some promising results have been reported. Specifically, preclinical studies have shown that alpha-lipoic acid improves mitochondrial function and reduces oxidative stress in the brain, which could be beneficial in neurodegenerative diseases [415,416,417,418]. Moreover, clinical trial studies using supplementation with alpha-lipoic acid have found that it improves cognition and mitochondrial function in patients with brain diseases [419,420,421]. Similarly, preclinical research has demonstrated that coenzyme Q10 can enhance mitochondrial function and protect brain cells from oxidative damage [410,422]. Additionally, coenzyme Q10 supplementation has shown potential benefits for cognitive function and slowing the progression of diseases such as Parkinson’s in clinical trials [423]. However, meta-analytical studies provided evidence that coenzyme Q10 was safe and well tolerated in people with Parkinson’s disease but not superior to placebo in terms of motor symptoms [424]. In the same way, preclinical studies have shown that NAD+ administration can improve mitochondrial function and protect against neurodegeneration (REF [425,426]. However, clinical studies using NAD+ in humans are still in their early stages, but promising results have been observed in managing neurodegenerative diseases [427]. Likewise, recently reviews have shown that creatine supplementation improves mitochondrial function and may have neuroprotective effects [428,429]. However, in humans, there are mixed results, with some studies finding some benefits in clinical outcomes [430] while others found no effect in the disease progression [431] after creatine supplementation.

Research evidence justifies the promising results of metabolic modulators due to several mechanisms of action to enhance mitochondrial function. First, metabolic modulators such as alpha-lipoic acid and coenzyme Q10 have antioxidant properties that reduce oxidative stress and protect mitochondria from damage [373,375,376]. Second, some metabolic modulators, like NAD+, can stimulate the production and maintenance of new mitochondria, thereby improving overall cellular function in the brain [408]. Third, metabolic modulators (e.g., coenzyme Q10) can facilitate electron transport in the mitochondrial respiratory chain, thereby improving ATP production and energy function [432]. Finally, certain metabolic modulators, like creatine, can regulate cellular metabolism, which may have beneficial effects on mitochondrial function and the protection of brain cells [433]. Therefore, together, preclinical and clinical studies suggest that metabolic modulators may be a promising strategy to improve mitochondrial function and manage brain diseases. However, further research is needed to fully understand their mechanisms of action and effectiveness in different neurological disorders.

## 14. Gene Therapy for Restoring Mitochondrial Function

Gene therapy has emerged as a promising approach for targeting mitochondrial dysfunction in the context of brain disease. By introducing therapeutic genes into affected cells, gene therapy aims to restore mitochondrial function and mitigate the underlying pathological mechanisms. Several studies have demonstrated the potential of gene therapy in preclinical models of brain disease, highlighting its efficacy in enhancing mitochondrial biogenesis, improving oxidative phosphorylation, and attenuating neuroinflammation [434,435]. One promising gene therapy strategy involves the delivery of genes encoding mitochondrial-targeted antioxidants, such as manganese superoxide dismutase (MnSOD) or catalase, to counteract the elevated reactive oxygen species (ROS) levels observed in brain disease [436]. Studies have shown that MnSOD gene delivery can reduce oxidative stress, preserve mitochondrial function, and ameliorate neurodegeneration in models of Parkinson’s disease [437]. Similarly, catalase gene therapy has been found to attenuate oxidative damage and improve cognitive function in models of Alzheimer’s disease [438].

Another gene therapy approach involves the delivery of genes encoding mitochondrial fusion proteins, such as mitofusin 1 and 2 (MFN1 and MFN2), to enhance mitochondrial dynamics and restore impaired mitochondrial networks. Restoration of mitochondrial fusion has been shown to ameliorate neuronal damage and improve motor function in animal models of neurodegenerative disorders [439]. In a study by Reddy and colleagues [436], adeno-associated viral vectors carrying MFN1 and MFN2 genes were intravenously administered to mice with Parkinson’s disease. The results demonstrated that restoration of mitochondrial fusion through overexpression of MFN1 and MFN2 improved mitochondrial function, reduced oxidative stress, and attenuated dopaminergic neuronal loss, leading to improved motor performance in the treated mice.

Furthermore, recent advancements in gene editing technologies, such as CRISPR-Cas9, have provided promising opportunities for correcting mitochondrial DNA (mtDNA) mutations associated with brain diseases. For instance, Gammage et al. [440] successfully utilised the CRISPR-Cas9 system to specifically target and correct a pathogenic mtDNA mutation in patient-derived fibroblasts [440]. This groundbreaking study showcased the potential of gene editing as a precise tool for correcting mtDNA mutations and restoring mitochondrial function. These findings highlight the therapeutic potential of gene therapies targeting mitochondrial dynamics and gene editing technologies for the treatment of neurodegenerative disorders and mtDNA-associated brain diseases, offering new possibilities for restoring mitochondrial function and improving patient outcomes.

Additionally, advancements in genome editing technologies, particularly CRISPR-Cas9, have opened new possibilities for correcting mitochondrial DNA (mtDNA) mutations associated with brain diseases. Several studies have demonstrated the successful use of CRISPR-Cas9 in editing mtDNA to correct pathogenic mutations in patient-derived cells, providing a promising avenue for the treatment of mitochondrial diseases [441]. This technology offers a simple design, low cost, high efficiency, and the ability to simultaneously edit multiple loci without the need for plasmids [442]. The potential of CRISPR-Cas9 in correcting mtDNA mutations highlights its significance in the field of gene editing and its application in addressing genetic abnormalities associated with mitochondrial dysfunction. The studies conducted by Tang et al. [443] and Ma et al. [444] provide evidence for the successful correction of pathogenic mtDNA mutations using CRISPR-Cas9 in patient-derived cells, reinforcing the potential of this technology for therapeutic interventions [441,443,444]

While gene therapy for mitochondrial dysfunction in brain disease is still in its early stages, these preclinical studies provide compelling evidence of its therapeutic potential. Further research is warranted to optimise gene delivery strategies, ensure long-term expression of therapeutic genes, and evaluate the safety and efficacy of gene therapy in clinical settings.

## 15. Conclusions and Future Perspectives: Challenges and Opportunities

In this culminating point, we delve into a comprehensive summary of the significant insights garnered throughout the review, shedding light on the intricate interplay between mitochondria and brain diseases. We acknowledge the existing challenges and illuminate the promising opportunities that lie ahead in this domain. Firstly, we address the knowledge gaps that persist in comprehending the multifaceted mechanisms underlying mitochondrial–brain pathology interactions. Despite remarkable progress, there is still much to explore and unravel regarding the precise molecular and cellular processes governing mitochondrial dysfunction in brain diseases. Further research endeavours are necessary to untangle these complexities and gain a deeper understanding of the underlying pathogenic mechanisms.

Next, we highlight the translational hurdles impeding the successful transition of mitochondrial-targeted therapies from preclinical investigations to clinical applications. Challenges encompass a range of factors, including effective drug delivery to specific brain regions, ensuring therapeutic specificity, and minimising potential side effects. Addressing these obstacles requires concerted efforts and innovative strategies to enhance treatment efficacy and safety. Moreover, we underscore the potential of emerging technologies, such as nanomedicine and gene editing, to revolutionise mitochondrial interventions for brain diseases. These cutting-edge approaches hold promise for precisely targeting and modulating mitochondrial function, providing new avenues for therapeutic advancements. Harnessing these technologies to refine and optimise mitochondrial-targeted therapies represents an exciting frontier with significant potential for clinical translation.

Furthermore, we emphasise the indispensability of interdisciplinary collaborations among researchers, clinicians, and industry partners. By fostering synergy among diverse expertise, we can accelerate progress in the fields of mitochondria and brain disease. Collaborative endeavours can propel the development of novel treatment modalities, facilitate knowledge sharing, and expedite the translation of scientific discoveries into tangible clinical benefits.

In conclusion, we paint an optimistic picture of the future, envisioning personalised and targeted treatments for brain diseases that leverage the therapeutic potential of mitochondria. By navigating the complexities, seizing the emerging opportunities, and fostering collaborative efforts, we can pave the way for transformative advancements in mitochondrial medicine. Ultimately, these strides will bring us closer to alleviating the burden of brain diseases and improving the lives of affected individuals.

## Data Availability

Not applicable.
